# Piezo1-ATF3-PPP1r15a Axis Transduces Mechanical Stress into Apoptosis in Glioma Under Low-Intensity Focused Ultrasound

**DOI:** 10.3390/cancers18091445

**Published:** 2026-04-30

**Authors:** Mingming Li, Weidong Wang, Jian Jiang, Yingxuan Mao, Mingwei Zhu, Linlin Han, Jiamei Niu, Pengfei Liu, Xiuhua Yang

**Affiliations:** 1 Department of Qunli Ultrasound, The First Affiliated Hospital of Harbin Medical University, Harbin 150001, China; zhongkao444@yeah.net (M.L.); duzun1001@163.com (W.W.); jimex1993@163.com (J.J.); my_x_ym@163.com (Y.M.); 815296@hrbmu.edu.cn (M.Z.); 18745004023@163.com (L.H.); 15776695250@163.com (J.N.); 2 Department of Magnetic Resonance, The First Affiliated Hospital of Harbin Medical University, Harbin 150001, China

**Keywords:** low-intensity focused ultrasound, endoplasmic reticulum stress, Piezo1, glioblastoma, apoptosis

## Abstract

Background: Glioblastoma (GBM) is characterized by high morbidity and mortality due to its localization and often locally invasive growth. Current treatment options for GBM are limited, with conventional therapies achieving a median survival of only 15 months. Mechanotherapy has been proposed as a new therapeutic strategy in oncology. Low-intensity focused ultrasound (LIFU), a form of mechanotherapy, has demonstrated inhibitory effects on GBM. However, its underlying mechanisms remain poorly understood. The present study aimed to evaluate the therapeutic effects of LIFU on GBM and investigate its mechanisms of action. Methods: Cell viability and proliferation were evaluated using cell counting kit-8, EdU and colony formation assays, while the effects of LIFU on GBM cell apoptosis were evaluated by flow cytometry. Transcriptome sequencing, immunofluorescence, reverse transcription-quantitative polymerase chain reaction, Western blot, bioinformatics analysis, dual-luciferase reporter assay and chromatin immunoprecipitation were used to investigate the molecular mechanisms underlying the effects of LIFU on GBM. The therapeutic efficacy of LIFU was further validated in a subcutaneous xenograft tumor model, in which tumor size, survival rate and immunohistochemical changes were monitored. Results: The results of the present study demonstrated that LIFU exerts anti-GBM effects by activating Piezo1 and modulating the downstream ATF3/PPP1r15a pathway to regulate apoptosis. LIFU therapy holds promise as a new treatment strategy for GBM, with the potential to improve patient prognosis. Conclusions: LIFU suppresses GBM progression through the Piezo1/ATF3/PPP1r15a axis by activating endoplasmic reticulum stress.

## Simple Summary

Glioblastoma is an aggressive brain tumor with limited treatment options and poor survival. Low-intensity focused ultrasound is a non-invasive therapy that uses gentle mechanical waves to target cancer cells without harming healthy tissue. In this study, we investigated how this ultrasound approach kills glioblastoma cells. We discovered that ultrasound activates a mechanical sensor called Piezo1 on the cell surface, which triggers a chain reaction involving the proteins ATF3 and PPP1r15a, ultimately leading to cancer cell death through endoplasmic reticulum stress. These findings reveal a new way to fight glioblastoma using mechanical forces. Understanding this pathway may help develop safer, more effective treatments for brain tumors and potentially other cancers, offering hope for improving patient outcomes.

## 1. Introduction

Glioblastoma (GBM) is the most common and aggressive malignant tumor of the central nervous system, characterized by a highly invasive growth pattern. Based on these statistics, the annual age-adjusted incidence rate of central nervous system tumors in the United States is 24.83/100,000 individuals [1]. Current standard treatments for GBM remain largely ineffective. The standard for adult-type diffuse gliomas begins with maximal safe surgical resection, followed by radiotherapy combined with chemotherapy. A recent study on glioblastoma surgery further underscores the central role of resection in the multimodal approach [2]. Molecularly targeted treatments continue to play only a secondary role in treatment strategies [3]. Immunotherapy for GBM is still in the early stages of development, with checkpoint inhibitor trials and vaccination studies failing to reveal enhanced outcomes in patients with GBM to date [4]. Emerging local treatment modalities, such as laser interstitial thermotherapy (LITT), have gained attention for their potential to achieve local tumor control, particularly in recurrent or deep-seated gliomas where repeat resection is challenging [5]. These clinical challenges underscore the urgent need to develop new, effective and low-toxicity therapeutic strategies for glioma, as informed by molecular biology studies.

Low-intensity focused ultrasound (LIFU) has emerged as a promising therapeutic modality owing to its exact targeting capability, which enables localized tumor treatment while sparing surrounding healthy tissues [6]. Unlike high-intensity focused ultrasound (HIFU), LIFU operates at low-intensity pulsed wave mode (acoustic intensity, 0–3 W/cm^2^; frequency, 100 kHz-3 MHz), delivering non-invasive mechanical stimulation to target tissues with minimal thermal effects [7]. Previous studies have demonstrated the antitumor efficacy of LIFU across different solid malignancies, including liver cancer [8], gastric cancer [9], cervical cancer [10] and breast cancer [11]. Notably, in vitro experiments demonstrated that LIFU can induce apoptosis in liver cancer cells through signaling pathways involving mitochondrial dysfunction and intracellular oxidative stress [12], thereby notably suppressing tumor growth.

Endoplasmic reticulum stress (ERS) increases from extracellular stressors or intracellular deoxyribonucleic acid (DNA) damage [13,14]. Emerging evidence suggests that ERS plays a key role in regulating gene expression and contributes to GBM pathogenesis [15]. During ERS, tumor cells activate the unfolded protein response (UPR), an adaptive mechanism that either restores ER homeostasis or triggers apoptosis when stress persists [16]. Upon disruption of proteostasis, UPR activation induces Ca^2+^ release from the ER, subsequently activating calcium/calmodulin-dependent protein kinase II activation and apoptotic signaling [17]. Among these mechanisms, modulating UPR effectors has emerged as a new therapeutic strategy for GBM. However, the capacity of LIFU to induce ERS in GBM remains unclear, as does the precise biophysical mechanism by which it complements conventional GBM treatments.

Discovered in 2010, Piezo channels represent a class of mammalian mechanosensitive ion channels that play crucial roles in transducing mechanical stimuli into cellular signals [18]. This family comprises two subtypes, namely Piezo1 and Piezo2 [19]. Piezo1 is uniquely activated by diverse mechanical stimuli, acting as a direct molecular transducer that changes physical forces, including pressure, shear stress and membrane stretch, into intracellular biochemical signals [20,21,22]. Functioning as a non-selective cation channel, Piezo1 activation triggers substantial Ca^2+^ influx, notably changing intracellular Ca^2+^ concentrations and consequently influencing cellular metabolism [23]. Notably, aberrant intracellular Ca^2+^ levels can induce cell death through multiple pathways. For instance, extracellular Ca^2+^ influx may cause ER calcium overload, leading to the accumulation of unfolded proteins and subsequent ERS initiation [24]. Despite these advances, the exact biophysical mechanisms through which Piezo1 contributes to GBM initiation and progression remain poorly understood.

In the present study, the transcriptome sequencing analysis of LIFU-treated GBM cells identified Piezo1 as a significantly upregulated gene compared with controls. Based on these findings, the following novel mechanotherapeutic paradigm was proposed: LIFU activates the Piezo1 ion channel to mediate extracellular Ca^2+^ influx, thereby triggering ERS and apoptosis in GBM cells. This mechanism may represent a broadly applicable therapeutic strategy for various tumor types. The therapeutic advantages of LIFU lie in its versatility and clinical feasibility. By precisely modulating ultrasound parameters (reducing and refining intensity), LIFU allows non-invasive tumor treatment without thermal ablation effects. This approach provides a safe and notable alternative for tumor treatment, combining targeted efficacy with minimal invasiveness.

## 2. Materials and Methods

### 2.1. Cell Culture

The American Type Culture Collection provided GBM cell lines (U251 and LN229) and the 293T cell line. All cell lines were incubated in DMEM (Gibco; Thermo Fisher Scientific, Inc., Waltham, MA, USA) containing 10% fetal bovine serum (Sigma-Aldrich, Burlington, MA, USA; Merck KGaA, Darmstadt, Germany) at 37 °C with 5% CO_2_, and were passaged every 48 h. The U251 and LN229 cell line used in this study was authenticated by Short Tandem Repeat (STR) profiling. The STR profiles were compared with the reference database ATCC, DSMZ, JCRB and RIKEN, and exhibited a 100% match. All cell lines used in this study were routinely tested for mycoplasma contamination. No mycoplasma contamination was detected in any of the experiments.

### 2.2. LIFU Operation

The LIFU machine was obtained from The Institute of Ultrasound of Chongqing Medical University (Chongqing, China), with an average intensity of SPTA 1 W/cm^2^. The transducer had a diameter of 2 cm, the focal length was 12.5 mm ± 15%, and the duty cycle was 50%. The coupling medium was ultrasound coupling gel. For all LIFU experiments, the control groups were sham-exposed controls (identical conditions without ultrasound irradiation).

### 2.3. Finite Element Analysis

The finite element model of the acoustic field was established using the commercial finite element software COMSOL Multiphysics 6.3 (COMSOL AB; Stockholm, Sweden). The water region was a hemisphere with a diameter of 50 mm, and the spherical wave radiation conditions were considered at the boundary of the water in the pressure-acoustic physics field. In the piezoelectric coupling physics, a voltage of 5 V was considered to excite the piezoelectric sheet; the thickness of the piezoelectric sheet was 2 mm, and the piezoelectric material was PZT-5H, which was polarized along the Z direction. The grid model had 40,919 cells and 20,699 nodes.

### 2.4. CCK-8 Assay

U251 and LN229 cells were seeded in 96-well plates at a density of 5000 cells/well. CCK-8 reagent (cat. no. IMC-906-5 mL; Xiamen Yimo Biotechnology Co., Ltd., Xiamen, China; 10 μL) was added to each well and incubated at 37 °C for 1.5 h. The absorbance was read at 450 nm using a microplate reader (BioTek; Agilent Technologies, Inc., Santa Clara, CA, USA).

### 2.5. Plate Colony Formation Assay

For the colony formation assay, a single-cell suspension of GBM cells (U251 and LN229) was prepared. Cells were seeded at a density of 1000 cells/well in 6-well plates and cultured in DMEM (Gibco; Thermo Fisher Scientific, Inc.) containing 10% fetal bovine serum (Sigma-Aldrich; Merck KGaA). Colonies were allowed to grow until they contained >50 cells. Subsequently, the cells were fixed with 4% paraformaldehyde for 15 min and stained with 0.1% crystal violet for 30 min at 37 °C. The result was photographed by a camera (Sony FX6; Sony Corporation, Tokyo, Japan). The number of colonies was counted using ImageJ software (1.54k). The colony formation rate was calculated as follows: (Number of colonies/number of seeded cells) × 100%.

### 2.6. Flow Cytometry Assay

Cells (U251 and LN229) were grown to ~80% confluence following LIFU intervention. According to the manufacturer’s protocol, Annexin V-FITC (Beyotime Biotechnology, Shanghai, China) was combined with the cells to be tested. The adherent cells were washed once with PBS and gently resuspended with 195 µL Annexin V-FITC binding solution. After which, 5 µL Annexin V-FITC and 10 µL PI staining solution were added, and the solution was mixed gently. The sample was incubated at room temperature (20–25 °C) in the dark for 10–20 min, followed by flow cytometry (BD FACSymphony A5; Becton, Dickinson and Company, Franklin Lakes, NJ, USA) detection. According to the manufacturer’s protocol, the cell cycle staining kit (cat. no. CCS012; Multi Sciences Biotech, Hangzhou, China) was combined with the cells to be tested, and 2 × 10^5^ cells were collected. The cells were washed once with PBS, then 1 mL DNA staining solution and 10 µL permeabilization solution were added, and the cells were incubated at room temperature in the dark for 30 min, followed by flow cytometry (BD FACSymphony A5; Becton, Dickinson and Company, Franklin Lakes, NJ, USA.) detection. The data were processed by FlowJo (Becton, Dickinson and Company, Franklin Lakes, NJ, USA)

### 2.7. Western Blot Assays

Cells (U251 and LN229) were lysed with RIPA buffer (cat. no. SW104; Suzhou Seven Biotechnology Co., Ltd., Suzhou, China) for 30 min, and then the cell lysate was scraped into an EP tube and centrifuged at 12,000× *g* at 4 °C for 10 min. The protein sample was mixed with loading buffer containing dithiothreitol and denatured by boiling for 10 min. The protein concentration was determined with the Enhanced BCA Protein Assay Kit (cat. no. P0010S; Beyotime Biotechnology) according to the manufacturer’s instructions. Mass of protein loaded per lane was 20 μg, sodium dodecyl sulfate-polyacrylamide gel electrophoresis separation was performed, percentage of gel was 10%, followed by transfer to an NC membrane and blocking with a rapid blocking agent (cat. no. P30500; Suzhou Xinsaimei Biotechnology Co., Ltd., Suzhou, China) for 15 min at room temperature. The membrane was incubated at 4 °C overnight with the following primary antibodies: Bcl-2 (1:1000, cat. no. 12789-1-AP; Proteintech Group, Inc., Rosemont, IL, USA), Bax (1:1000, cat. no. 50599-2-Ig; Proteintech Group, Inc.), caspase3 (1:1000, cat. no. 19677-1-AP; Proteintech Group, Inc.), ATF3 (1:500, cat. no. YT0387; ImmunoWay Biotechnology Company, Plano, TX, USA), ATF4 (1:1000, cat. no. 81798-1-RR; Proteintech Group, Inc.), CHOP (1:1000, cat. no. 81462-1-RR; Proteintech Group, Inc.), EIF2α (1:1000, cat. no. 82936-1-RR; Proteintech Group, Inc.), p-EIF2α (1:2000, cat. no. 87179-2-RR; Proteintech Group, Inc.), β-actin (1:50,000, cat. no. 66009-1-Ig; Proteintech Group, Inc.) and PPP1r15a (1:1000, cat. no. a16260; ABclonal Technology Co., Ltd., Woburn, MA, USA). After washing three times with PBST, the membrane was incubated for 1 h at room temperature with a fluorescent secondary antibody IRDye 680RD Goat anti-Mouse IgG (cat. no. 926-68070; LI-COR, Inc., Lincoln, NE, USA). Finally, the Odyssey developer (ODYSSEY^®^ DLx; LI-COR, Inc.) was used to develop the image. Protein band densities from Western blots were quantified using ImageJ software (National Institutes of Health).

### 2.8. EdU Cell Proliferation Assay

According to the manufacturer’s protocol, following the treatment period, an equal volume of EdU working solution (20 μM) was added to each well of a 6-well plate, and the cells were incubated for 2 h. After fixation and permeabilization, the Click reaction cocktail (0.5 mL/well) was applied, and the plates were incubated in the dark at room temperature for 30 min. Finally, cell nuclei were counterstained with Hoechst 33342 at room temperature for 10 min, and images were acquired using a confocal microscope.

### 2.9. RNA Sequencing

U251 cells were exposed to LIFU (1 W/cm^2^) for 40 s, followed by three bioduplications. Total RNA was extracted using a Trizol kit (cat. no. CW0580; Beijing CWBIO Co., Ltd., Beijing, China), and RNA sequencing was performed by Beijing Novogene Technology Co., Ltd. (Beijing, China) using the Fast RNA-seq Lib Prep Kit V2, and Illumina NovaseqX Plus. RNA integrity was assessed using the Bioanalyzer 2100 system (Agilent Technologies, Inc.). After Illumina Casava (1.8) base calling, raw data (fastq) were processed through fastp software (v0.23.4). Differential expression analysis was performed on biological replicates for two conditions/groups using the DESeq2 R package (1.20.0). DESeq2 provides statistical programs for determining differential expression in digital gene expression data using models based on negative binomial distribution. The resulting *p*-value was adjusted using Benjamini and Hochberg’s methods to control the error discovery rate. The corrected *p* ≤ 0.05 and |log2(foldchange)| > 0.5 was set as the threshold of significant differential expression. Gene Ontology (GO) and Kyoto encyclopedia of genes and genomes (KEGG) enrichment analysis of differentially expressed genes was implemented by the clusterProfiler R package, in which gene length bias was corrected. PPI analysis of differentially expressed genes was based on the STRING database.

### 2.10. Reverse Transcription-Quantitative PCR

Total RNA was extracted from adherent U251 or LN229 cells using the SevenFast^®^ kit (cat. no. SM130; Suzhou Seven Biotechnology Co., Ltd.) according to the manufacturer’s protocol. Briefly, after removal of the culture supernatant, cells were lysed directly in the culture dish by adding 500 μL SevenFast^®^ Lysis Buffer and pipetting vigorously with an RNase-free tip. The lysate was incubated at room temperature for 3 min for complete dissociation. After which, the homogenate was transferred to a collection column and centrifuged at 12,000× *g* for 30 s. The flow-through was discarded, and the column membrane was washed twice with 500 μL Wash Buffer (12,000× *g*, 30 s/wash). To remove residual ethanol, the column was centrifuged at 12,000× *g* for 2 min at 4 °C. Purified RNA was eluted by applying 30–50 μL nuclease-free water to the membrane center, incubating for 1 min at room temperature, and centrifuging at 10,000× *g* for 1 min at 4 °C. Complementary DNA was synthesized by reverse transcription using a commercial kit (cat. no. FSQ-301; Toyobo Co., Ltd., Tokyo, Japan) according to the manufacturer’s instructions. The reverse transcription reaction was assembled in a final volume of 10 μL containing 1 μg total RNA template, 2 μL 4× DN Master Mix, 2 μL 5× RT Master Mix II and nuclease-free water. The mixture was gently tapped and briefly centrifuged. Reverse transcription was performed under the following thermal conditions: 37 °C for 15 min, 50 °C for 5 min and 98 °C for 5 min for enzyme inactivation, followed by a hold at 4 °C. All primers are listed in Table S1. The system was prepared with sangon 1× SYBR Green qPCR Master Mix (cat. no. B532955; Sangon Biotech (Shanghai) Co., Ltd., Shanghai, China) and qPCR was performed using the following amplification program: 95 °C for 5 min, 95 °C for 10 s, 60 °C for 30 s, for 40 cycles. GAPDH was used as the normalization reference, and the relative mRNA level of the target gene was calculated using the 2^−ΔΔCq^ method [25].

### 2.11. Immunofluorescence

Cells were washed with PBS and fixed with 4% paraformaldehyde at room temperature for 20 min. Depending on the protein target, a tailored protocol was applied. Nuclear antigen detection required permeabilization with 3% Triton X-100 prior to blocking with goat serum (cat. no. E-IR-R110; Elabscience Biotechnology Co., Ltd., Wuhan, China) at room temperature for 1 h and subsequent incubation with the primary antibody against ATF3 (1:100; cat. no. YT0387; ImmunoWay Biotechnology Company). By contrast, for membrane protein detection, cells were directly blocked with goat serum at room temperature for 1 h before overnight incubation at 4 °C with the primary antibody against Piezo1 (1:50; cat. no. 15939-1-AP; Proteintech Group, Inc.). Following three washes with PBS, the cells were incubated with a fluorescent secondary antibody (1:100; ABclonal Biotech Co., Ltd.; cat. no. AS 011) at room temperature for 1 h. After incubation, the cells were washed three times with PBST for 5 min, counterstained with DAPI for 10 min at room temperature, and then washed again three times with PBST for 5 min. Finally, the samples were mounted with an anti-fade mounting medium and visualized using a FLUOVIEW FV3000 confocal laser scanning microscope (Olympus Corporation, Tokyo, Japan).

### 2.12. Small-Interfering RNA (siRNA) Transfection

Cell transfection was performed using Lipofectamine 2000^®^ (Thermo Fisher Scientific, Inc.). SiRNA was designed by Sangon Biotech (Shanghai) Co., Ltd. For each well of a six-well plate, 3 μL siRNA (sequences in Table S1) and 5 μL Lipofectamine 2000^®^ were separately diluted in 250 μL Opti-MEM Reduced Serum Medium. Final siRNA concentration was 100 nM. After a 5 min incubation at room temperature, the two solutions were combined, and then added to cells previously rinsed with serum-free medium. Following 6 h of incubation at 37 °C, the transfection mixture was replaced with complete culture medium. Following experiments were started after 24 h.

### 2.13. Mitochondrial Membrane Potential Assay (JC-1)

The culture medium was aspirated, and the cells were washed once with PBS. Subsequently, 1 mL fresh culture medium was added, followed by the addition of 1 mL JC-1 staining working solution, which was gently mixed to ensure even distribution. After which, the cells were incubated at 37 °C in a CO_2_ incubator for 20 min. Following incubation, the supernatant was carefully removed, and the cells were washed twice with the provided JC-1 staining buffer. Finally, 2 mL DMEM (Gibco; Thermo Fisher Scientific, Inc.) containing 10% fetal bovine serum (Sigma-Aldrich; Merck KGaA) was added, and the cells were immediately observed under a laser scanning confocal microscope.

### 2.14. Bioinformatics Analysis

The Venn diagram was generated using the Novogene Cloud Platform (https://magic-plus.novogene.com/#/ (accessed on 15 October 2024)). The potential protein–protein interaction between ATF3 and PPP1r15a was assessed using the STRING database (https://string-db.org/ (accessed on 15 October 2024)), and the functional association network between ATF3 and PPP1r15a was analyzed using the GeneMANIA database (https://genemania.org/ (accessed on 19 October 2024)). Potential ATF3-binding sites in the PPP1r15a promoter region were predicted using the JASPAR database (https://jaspar.elixir.no/ (accessed on 19 December 2024)). Pearson correlation analysis was performed to assess the correlation between ATF3 and PPP1r15a expression in the TCGA glioma dataset using UALCAN (https://ualcan.path.uab.edu/index.html (accessed on 23 October 2024)). Statistical significance for analyses conducted using the UALCAN and GeneMANIA databases was defined as a false discovery rate (FDR; Benjamini–Hochberg-corrected *p*-value) of <0.05. For the STRING database, only interactions with a combined confidence score > 0.70 were retrieved. Putative transcription factor binding sites in the JASPAR database were selected using a relative profile score threshold of ≥85% of the highest possible score.

### 2.15. Dual-Luciferase Reporter Assay

Cell transfection was performed using Lipofectamine 2000^®^ (Thermo Fisher Scientific, Inc.). U251 siATF3 cells or 293T cells were seeded in the 24-well plate 24 h before transfection. Once the cells reached 80–90% confluency, pGL3-basic-PPP1r15a promoter (Shanghai GenePharma Co., Ltd., Shanghai, China) and pGL3-basic-PPP1r15a promoter mutation (Wuhan Jinkairui Bioengineering Co., Ltd., Wuhan, China) constructs were transfected with the ATF3 overexpression plasmid (Shanghai GenePharma Co., Ltd.), and pRL-TK (Shanghai GenePharma Co., Ltd.) was also transfected to normalize the transfection efficiency. All plasmid sequences in Table S2. The ratio of promoter to overexpression plasmid to pRT-TK was 100:100:1. The cells were harvested 48 h post-transfection, and the luciferase activity was immediately measured using a Dual-Luciferase reporter assay kit (cat. no. FR201-02-V2; Beijing TransGen Biotech Co., Ltd., Beijing, China).

### 2.16. ChIP

Cells in a 10 cm dish were cross-linked by adding 270 µL 37% formaldehyde, and the reaction was quenched with glycine solution (10×). After washing with cold PBS containing protease inhibitors, cells were scraped and collected. Chromatin was sheared on ice by sonication (10 s on/10 s off; 20 cycles) with a frequency of 20 W to generate fragments of appropriate size. Immunoprecipitation was conducted as described previously [26]. Chromatin samples were subjected to immunoprecipitation by incubation with the ATF3 antibody (Cat. No. 18665T; Cell Signaling Technology, Inc., Danvers, MA, USA) overnight at 4 °C with gentle rotation. Subsequently, 80 µL Protein A/G Magnetic Beads pre-blocked with salmon sperm DNA were added, and incubation continued for 60 min at 4 °C. The bead complexes were washed sequentially once each with Low Salt, High Salt and LiCl Immune Complex Wash Buffers, followed by two washes with TE Buffer. To reverse cross-links, 20 µL 5 M NaCl was added, and the samples were incubated at 65 °C for 4 h. DNA was then purified by phenol-chloroform extraction. Enrichment of the PPP1r15a promoter region was finally analyzed by qPCR. Normal IgG was used as a negative control to assess non-specific background. ChIP data were calculated and presented as % of Input.

### 2.17. Intracellular Calcium Measurement

The culture medium was aspirated, and the cells were gently washed three times with PBS. Subsequently, 1 mL Fluo-4 AM working solution was added, followed by incubation at 37 °C for 60 min to load the dye. After loading, the cells were washed three times with PBS to terminate the loading process. Hochest 3342 stained the nucleus, followed by fluorescence imaging. In experiments using GsMTx4 or Yoda1, control groups received an equal volume of the respective vehicle (normal saline for GsMTx4; DMSO for Yoda1).

### 2.18. Construction of a Xenograft Tumor Model

10 female and 10 male BALB/c nude mice (4–5 weeks, 18–20 g) were obtained from Jiangsu Huachuang Cigna Pharmaceutical Technology Co., Ltd. (Huai’an, China), [license no. SCXK(Su)2020-0009]. The mice were housed under conventional conditions (room temperature 22 ± 1 °C, humidity 50 ± 5% and 12/12 h light/dark cycle) with free access to food and water. Animal experiments were approved by The Animal Welfare Ethics Committee of the First Affiliated Hospital of Harbin Medical University (Harbin, China; approval no. 2024056). U251 cells (1 × 10^6^/100 μL) in the mice were resuspended in PBS and inoculated under the armpit until the tumor volume reached 50 mm^3^. A total of eight days after U251 cell injection, the mice were randomly divided into the following four groups: (i) Control (*n* = 5); (ii) LIFU (*n* = 5); (iii) GsMTx4 + LIFU (*n* = 5); and (iv) 4-PBA + LIFU (*n* = 5). The inhibitors 4-phenylbutyric acid (4-PBA) and GsMTx4 were administered prior to LIFU intervention. Specifically, 4-PBA was dissolved in normal saline and injected intraperitoneally at a daily dose of 150 mg/kg, and GsMTx4 was delivered via local injection at a daily dose of 10 µg/kg. Tumor size (tumor volume = length × width^2^/2) was measured daily with a vernier caliper, following continuous irradiation once every two days for 14 days. The animals were euthanized by cervical dislocation, after which the tumors were dissected, imaged and weighed. The outcome assessment was conducted by an investigator who was blinded to the group allocation to minimize bias.

### 2.19. Immunohistochemistry (IHC)

Tissues were fixed in 4% paraformaldehyde at 4 °C for 48 h. Paraffin sections were cut at 4 μm thickness. After the paraffin sections were dewaxed in xylene, the sections were rehydrated with gradient ethanol. Tissue sections were subjected to antigen retrieval using EDTA antigen retrieval buffer at 100 °C, and 3% H_2_O_2_ solution was injected to block endogenous peroxidase activity. Following permeabilization with 0.04% Triton X-100 for 20 min at room temperature, the sections were blocked with 10% goat serum (Beijing Zhongshan Jinqiao Biotechnology Co., Ltd., Beijing, China; cat. no. ZLI-9022) for 60 min at room temperature. Following incubation at 37 °C overnight with primary antibodies: ATF3 (1:100, cat. no. YT0387; ImmunoWay Biotechnology Company), ATF4 (1:50, cat. no. 81798-1-RR; Proteintech Group, Inc.), CHOP (1:50, cat. no. 15204-1-AP; Proteintech Group, Inc.), the sections were incubated with horseradish peroxidase-conjugated goat anti-rabbit IgG (1:2000; Wuhan Servicebio Technology Co., Ltd., Wuhan, China; cat. no. GB23204) at 37 °C for 2 h. Subsequently, the sections were incubated with biotinylated secondary antibodies (1:2000; Zhongshan Jinqiao Biotechnology Co., Ltd., cat. no. PV-9001) at room temperature for 2 h. Color development was performed using a 3,3′-diaminobenzidine substrate kit (Zhongshan Jinqiao Biotechnology Co., Ltd., cat. no. ZLI-9017), and the stained sections were observed under a light microscope. Sections scanning and analysis were performed by KFSlideOS (Wuhan Baiqiandu Biotechnology Co., Ltd., Wuhan, China)

### 2.20. Statistical Analysis

Data are presented as the mean ± standard error of the mean from at least three independent biological replicates. Statistical analyses were performed using GraphPad Prism software 10.1.2 (Dotmatics). For comparisons between two groups, a two-tailed unpaired Student’s *t*-test was used when data met assumptions of normality and equal variance. If variances were unequal (as determined by an F-test), the Welch *t*-test was applied. For comparisons among multiple groups with a single independent variable, one-way ANOVA was conducted. Provided normality was satisfied, Tukey’s post hoc test was used for multiple comparisons. If the assumption of equal variance was violated (assessed by the Brown-Forsythe test), a Brown-Forsythe and Welch ANOVA with Dunnett’s T3 post hoc test was employed. For comparisons involving more than two independent variables, two-way ANOVA followed by Šídák’s multiple comparisons test was performed. *p* < 0.05 was considered to indicate a statistically significant difference. For the xenograft study, Post hoc power analysis using the observed effect size on tumor volume indicated a power of 0.99 (α = 0.05), confirming that the study was adequately powered to detect significant differences.

## 3. Results

### 3.1. LIFU Exerted Potent Antitumor Effects

**Figure 1:**
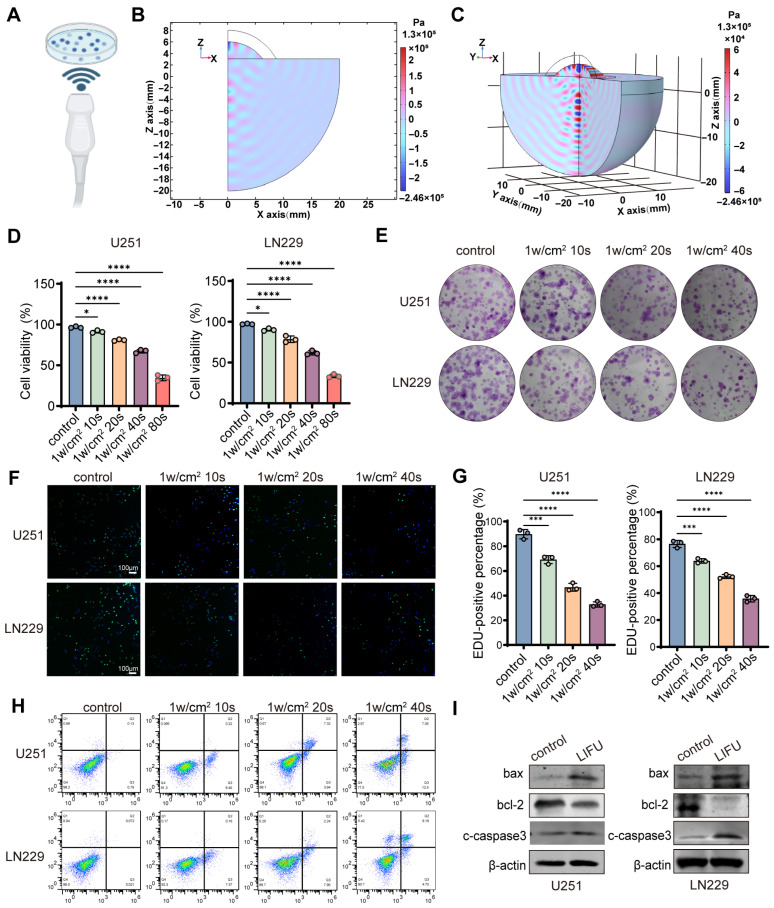
LIFU exerts potent antitumor effects. (**A**) LIFU intervention diagram (Dark blue represents cells, and light blue represents the culture medium). (**B**) The two-dimensional sound field distribution was displayed with FEM analysis. (**C**) The three-dimensional sound field distribution was displayed with FEM analysis. (**D**) Cell viability of U251 and LN229 cells treated with LIFU. (**E**) Colony formation assays were performed after U251 and LN229 cells were treated with LIFU. (**F**) Representative images of EdU in U251 and LN229 cells with different treatments. (**G**) The corresponding statistical results of the EdU-positive percentage. (**H**) The percentages of annexin-V/PI bound cells. (**I**) Bax, Bcl-2 and caspase3 levels were determined by Western blot. The data are presented as the means ± SEM from at least three independent biological replicates. Statistical comparisons among multiple groups were performed using one-way ANOVA followed by Tukey’s post hoc test (* *p* < 0.05; *** *p* < 0.001; **** *p* < 0.0001; *n* = 3). FEM, finite element method; LIFU, low-intensity focused ultrasound. The original Western blot figures can be found in Supplementary File S1.

As illustrated in Figure 1A, the schematic demonstrates the LIFU irradiation setup where cells in culture dishes were exposed to ultrasound waves propagating upward from beneath. The acoustic field generated by LIFU was simulated by finite element modeling. At an excitation voltage of 5 V, the maximum acoustic pressure was ~0.1 MPa. The surface acoustic pressure had alternating positive and negative phases with progressive attenuation along the propagation depth (Figure 1B,C and Figure S1A,B). Concurrently, temperature changes at the focal point of the focused ultrasound were monitored in real-time using a FLIR thermal imaging camera. The measurements revealed a temperature increase of 0.3 ± 0.1 °C at the focal region in the LIFU group compared with the control (Figure S1C,D). CCK-8 assays were performed at an intensity of 1 W/cm^2^ with varying exposure durations including 0 s, 10 s, 20 s, 40 s, and 80 s. The findings demonstrated a time-dependent, significant decrease in cell viability for U251 and LN229 cell lines after LIFU treatment (Figure 1D). We treated U251 and LN229 GBM cells with a fixed exposure duration of 40 s and varied the LIFU intensity (0, 0.5, 1.0, and 2.0 W/cm^2^). Cell viability was then assessed using the CCK-8 assay. The results demonstrate a clear intensity-dependent reduction in cell viability in both cell lines, with higher intensities leading to progressively greater cytotoxicity (Figure S1E). Although 1 W/cm^2^ 80 s exhibited stronger tumor inhibitory effects, from a translational medicine perspective, the 1 W/cm^2^ 40 s regimen offers a more favorable balance between efficacy and anticipated safety, making it more suitable for potential clinical applications. Therefore, we selected 1 W/cm^2^ for 40 s as the LIFU treatment condition for all subsequent experiments. The parameters chosen for this experiment are in line with those reported in prior work [27]. To rule out the possibility that apoptosis was caused by physical detachment following LIFU irradiation, we performed trypan blue staining. The results showed a slight increase in the number of detached cells in the LIFU group compared to the control group (Figure S1F), accounting for only about 3% of the total cell population. Among the detached cells, approximately 86% in the control group were trypan blue negative, whereas about 68% in the LIFU group were trypan blue positive (Figure S1F), indicating that the majority of detached cells in the LIFU group were apoptotic. These findings demonstrate that the observed tumor inhibitory effect is attributable to apoptosis directly induced by LIFU, rather than physical detachment. Colony formation assays indicated a marked reduction in colony numbers in the 1 W/cm^2^, 40 s LIFU treatment group (Figure 1E and Figure S1G). The EdU assays confirmed the suppression of cell proliferation (Figure 1F,G). Flow cytometric analysis using Annexin V-FITC/PI staining demonstrated that LIFU treatment induced apoptosis in GBM cells (Figure 1H and Figure S1H). We have performed additional CCK-8 assays to evaluate the cytotoxicity of LIFU on normal cells. Specifically, we treated normal HT22 with the same LIFU parameters (1 W/cm^2^, 40 s) applied to glioblastoma cells. The results clearly demonstrated that LIFU did not significantly reduce the viability of normal cells compared to untreated controls (Figure S1I). Western blot analysis demonstrated the variation in the apoptosis marker, and these molecular alterations consistently assisted the observed phenotypic alterations (Figure 1I and Figure S1J).

### 3.2. ATF3 Was Induced in Response to LIFU Treatment

**Figure 2:**
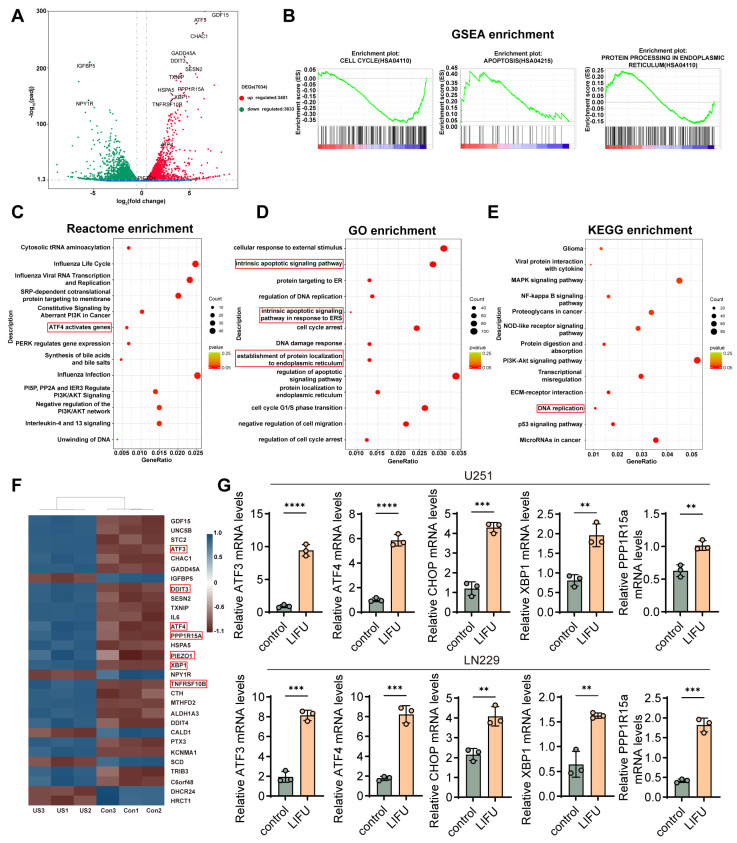
ATF3 is induced in response to LIFU treatment. (**A**) The differential gene expression among control and LIFU groups (volcano plot). (**B**) GSEA in the control group and LIFU group. (**C**) Reactome enrichment analyses with the DEGs (The red box represents pathways related to endoplasmic reticulum stress.). (**D**) GO enrichment analyses with the DEGs (The red box denotes the pathways related to endoplasmic reticulum stress and apoptosis). (**E**) KEGG enrichment analyses with the DEGs (The red box indicates pathways associated with apoptosis). (**F**) The differential gene expression among control and LIFU groups (heatmap), the red box indicates genes related to endoplasmic reticulum stress. (**G**) U251 and LN229 cells were treated with LIFU and subjected to reverse transcription-quantitative PCR analysis for CHOP, ATF3, ATF4, XBP1 and PPP1r15a. The mRNA levels of CHOP, ATF3, ATF4 and PPP1r15a were normalized to those of GAPDH. The data are presented as the means ± SEM from at least three independent biological replicates. ** *p* < 0.01; *** *p* < 0.001; **** *p* < 0.0001. LIFU, low-intensity focused ultrasound; GSEA, Gene Set Enrichment Analysis; GO, Gene Ontology; KEGG, Kyoto Encyclopedia of Genes and Genomes; DEGs, differentially expressed genes.

To elucidate the mechanism underlying LIFU-mediated inhibition of GBM development, RNA sequencing on GBM cells was performed and differential gene expression following LIFU intervention was evaluated. Differentially expressed genes were identified using the criteria of |log2FC| > 0.5 and *p* < 0.05. Transcriptome sequencing revealed that LIFU treatment of U251 cells resulted in 3401 upregulated and 3633 downregulated genes (Figure 2A,F). Subsequent GO, Reactome and KEGG analyses demonstrated promising enrichment in the ERS signaling pathway (Figure 2C–E), with ATF3 being the most differentially expressed gene in this pathway. Gene set enrichment analysis (GSEA) indicated improved pathways associated with ERS, cell cycle arrest and apoptosis in U251 cells (Figure 2B). While cells initially ensure ERS homeostasis for survival, excessive ERS can ultimately induce cell death. To determine whether these effects were consistent across various GBM cell lines, the expressions of CHOP, ATF3, XBP1, PPP1r15a and ATF4 were examined in two GBM cell models. Notably, all five genes indicated significantly increased expression following LIFU stimulation (Figure 2G). Western blot analysis was performed to examine the protein expression of ATF3, ATF4 and CHOP. The results demonstrated that LIFU treatment significantly increased the expression of ATF3, ATF4 and CHOP, suggesting ERS activation (Figure S2A,B). In summary, the present sequencing findings indicate that LIFU may establish GBM cell apoptosis through activation of the ERS pathway.

### 3.3. Piezo1 Mediated LIFU-Induced ERS Effects

**Figure 3:**
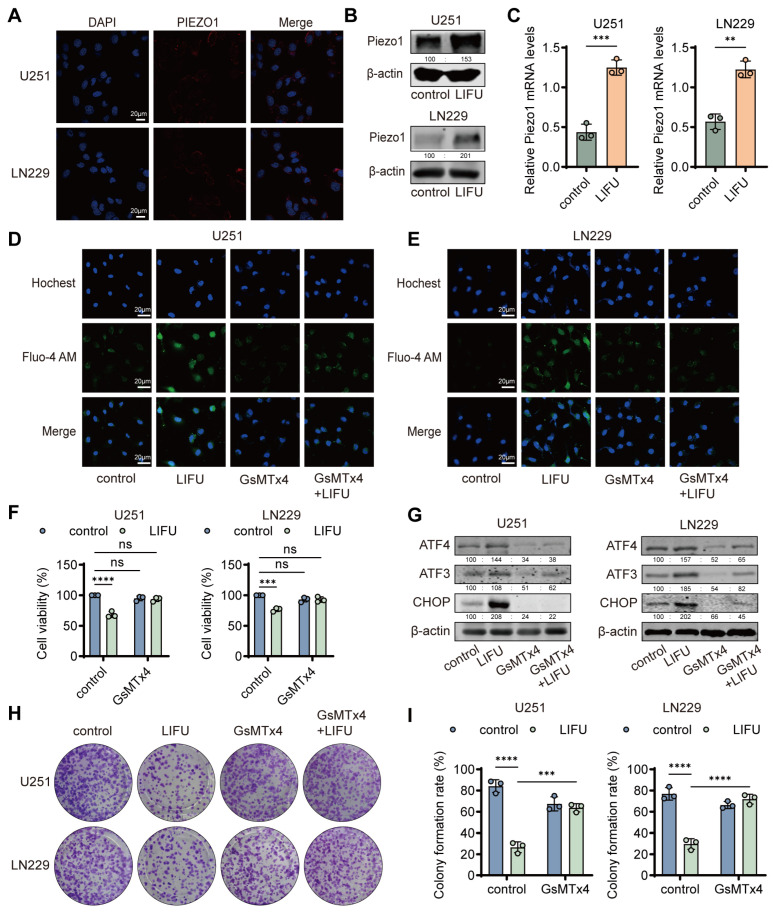
Piezo1 mediates LIFU-induced ERS effects. (**A**) Representative IF images of Piezo1. (**B**) Piezo1 expression levels were determined by Western blot. (**C**) U251 and LN229 cells were treated with LIFU and then subjected to reverse transcription-quantitative PCR analysis for Piezo1. The mRNA levels of Piezo1 were normalized to those of GAPDH. (**D**) Intracellular calcium levels were labeled by Fluo-4 (green) after different interventions in U251. (**E**) Intracellular calcium levels were labeled by Fluo-4 (green) after different interventions in LN229. (**F**) Cell viability of U251 and LN229 cells treated with LIFU. (**G**) ATF4, ATF3 and CHOP were determined by Western blot. (**H**) Colony formation assays were performed in the control, LIFU, GsMTx4 or LIFU + GsMTx4 groups. (**I**) The corresponding statistical results of the colony formation rate. The data are presented as the means ± SEM from at least three independent biological replicates. Statistical comparisons among multiple groups (control, LIFU, GsMTx4, LIFU + GsMTx4) were performed using two-way ANOVA followed by Šídák’s multiple comparisons test. ** *p* < 0.01; *** *p* < 0.001; **** *p* < 0.0001. LIFU, low-intensity focused ultrasound; ERS, endoplasmic reticulum stress; IF, immunofluorescence. The original Western blot figures can be found in Supplementary File S1.

Given that LIFU is a form of mechanical stimulation, we sought to identify upstream molecules involved in sensing this mechanical signal. Piezo1, a mechanosensitive calcium channel localized on the plasma membrane (Figure 3A), has been reported to be activated by LIFU. The RNA sequencing in the present study demonstrated upregulated Piezo1 mRNA expression in LIFU-treated GBM cells (Figure 2F). Both qPCR and Western blot confirmed significantly higher Piezo1 expression in LIFU-treated cells (Figure 3B,C and Figure S1K). The fluo-4 AM staining demonstrated improved Ca^2+^ influx post-LIFU. This effect was abolished by the specific Piezo1 inhibitor, GsMTx4 (Figure 3D,E and Figure S3A). The EdU assays indicated that GsMTx4 partially rescued LIFU-induced growth inhibition (Figure S2C,D). CCK-8 assays revealed decreased apoptosis with GsMTx4 co-treatment (Figure 3F). Mechanistically, Western blot analyses confirmed that GsMTx4 effectively suppressed ERS by LIFU (Figure 3G and Figure S2E,F). Notably, in the LIFU treatment alone compared with the GsMTx4 + LIFU co-treatment group, the colony formation rate was significantly attenuated (Figure 3H,I). SiPiezo1 (Table S3) was designed and successfully transfected, with knockdown efficiency confirmed by PCR and immunofluorescence (Figure S3B,C). Using the most effective siRNA (siPiezo1#3), it was observed that Piezo1 knockdown markedly attenuated LIFU-induced ERS (Figure S3D,E). Subsequently, we treated GBM cells with the specific Piezo1 agonist Yoda1 (10 µM). Consistent with the results obtained with LIFU, Yoda1 treatment significantly enhanced apoptosis in GBM cells (Figure S3F). Western blot analysis further revealed that Yoda1 markedly upregulated ATF3 protein levels (Figure S3G). Yoda1 treatment led to a significant increase in Ca^2+^ influx compared to the control (Figure S4A,B). Collectively, these results provide direct evidence that activation of Piezo1 alone phenocopies the effects of LIFU-inducing Ca^2+^ influx, ATF3 upregulation and apoptosis. Together with our Piezo1 knockdown experiments, these findings establish that Piezo1 is necessary for driving the LIFU-induced signaling cascade in GBM cells.

### 3.4. ATF3 Was Involved in the ERS of GBM Cells Treated with LIFU

**Figure 4:**
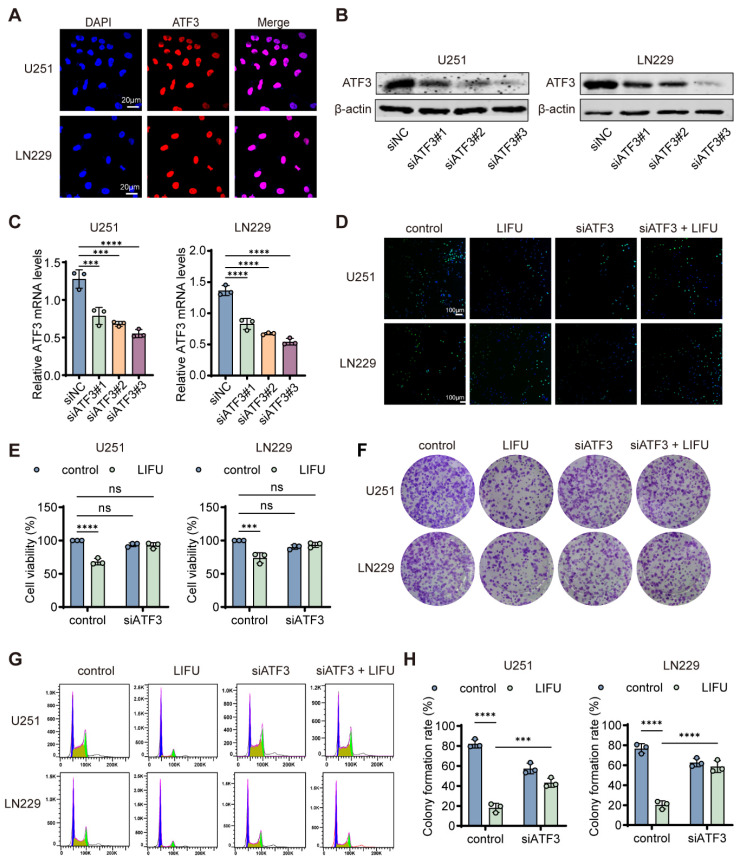
ATF3 is essential for LIFU. (**A**) Representative IF images of ATF3. (**B**) ATF3 expression was determined by Western blot. (**C**) Relative ATF3 mRNA levels of siATF3. (**D**) Representative images of EdU in U251 and LN229 with different treatments. (**E**) Cell viability of U251 and LN229 cells. (**F**) Colony formation assays were performed with different treatments. (**G**) Cell cycle was performed with different treatments (purple peak: G1 phase; green peak: G2 phase; yellow region between G1 and G2: S phase). (**H**) The corresponding statistical results of the colony formation. The data are presented as the means ± SEM from at least three independent biological replicates. Statistical comparisons among multiple groups (control, LIFU, siATF3, LIFU + siATF3) were performed using two-way ANOVA followed by Šídák’s multiple comparisons test. *** *p* < 0.001; **** *p* < 0.0001. LIFU, low-intensity focused ultrasound. The original Western blot figures can be found in Supplementary File S1.

**Figure 5:**
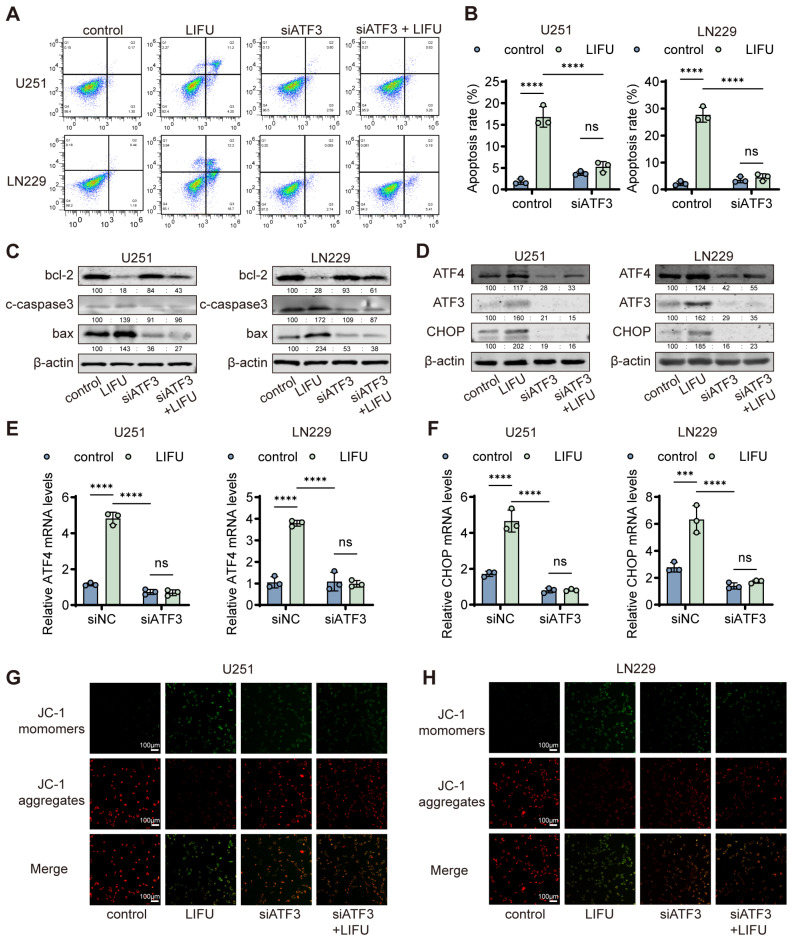
ATF3 is involved in the ERS of GBM cells treated with LIFU. (**A**) Annexin-V/PI bound U251 or LN229 cells were counted. (**B**) The corresponding statistical results of apoptosis rate. (**C**) The expression levels of Bax, Bcl-2 and caspase3 were determined by Western blot. (**D**) The expression levels of ATF3, ATF4 and CHOP were determined by Western blot. (**E**) U251 and LN229 cells were subjected to reverse transcription-quantitative PCR analysis for ATF4. (**F**) U251 and LN229 cells were subjected to reverse transcription-quantitative PCR analysis for CHOP. (**G**) Representative images of JC-1 staining in U251 with different treatments. (**H**) Representative images of JC-1 staining in LN229 with different treatments. The data are presented as the means ± SEM from at least three independent biological replicates. Statistical comparisons among multiple groups (control, LIFU, siATF3, LIFU + siATF3) were performed using two-way ANOVA followed by Šídák’s multiple comparisons test. *** *p* < 0.001; **** *p* < 0.0001. ER, endoplasmic reticulum; GBM, glioblastoma; LIFU, low-intensity focused ultrasound. The original Western blot figures can be found in Supplementary File S1.

Further exploring the role of ATF3 in LIFU-mediated apoptosis, immunofluorescence demonstrated nuclear localization of ATF3 (Figure 4A). After which, siATF3 (Table S3) was designed and successfully transfected, with knockdown efficiency confirmed by PCR and Western blot (Figure 4B,C and Figure S4C). Using the more effective siRNA (siATF3#2 and siATF3#3), it was observed that ATF3 knockdown markedly attenuated LIFU-induced cytotoxicity, as evidenced by increased cell viability in CCK-8 assays (Figure 4E). EdU (Figure 4D and Figure S4D), colony formation (Figure 4F,H and Figure S6A,B) assays and flow cytometric analysis using Annexin V-FITC/PI staining (Figure 5A,B and Figure S6C,D) confirmed that ATF3 depletion partially rescued the anti-proliferative effects of LIFU. Flow cytometry revealed LIFU-induced G0/G1 cell cycle arrest and S phase reduction (Figure 4G and Figure S4E,F). In the four experimental groups (control, siNC + LIFU, siATF3 and siATF3 + LIFU), Western blot indicated that ATF3 knockdown reversed LIFU-induced apoptotic changes (Figure 5C and Figure S5A,B). Molecular analysis indicated that ATF3 silencing reduced CHOP and ATF4 expression (Figure 5D–F and Figure S5C,D). Mechanistically, JC-1 staining revealed that LIFU decreased mitochondrial membrane potential, which was partially restored using ATF3 knockdown (Figure 5G,H). Collectively, these results establish ATF3 as a crucial mediator of LIFU-induced ERS and subsequent apoptosis in GBM cells. We used the ER stress inhibitor 4-PBA to validate the ER stress response induced by LIFU in vitro. Western blot results showed that 4-PBA significantly inhibited the LIFU-induced increase in p-eIF2α levels (Figure S6E,F).

### 3.5. ATF3 Directly Bound to the PPP1r15a Promoter and Enhanced the Transcriptional Activity of the PPP1r15a Promoter

**Figure 6:**
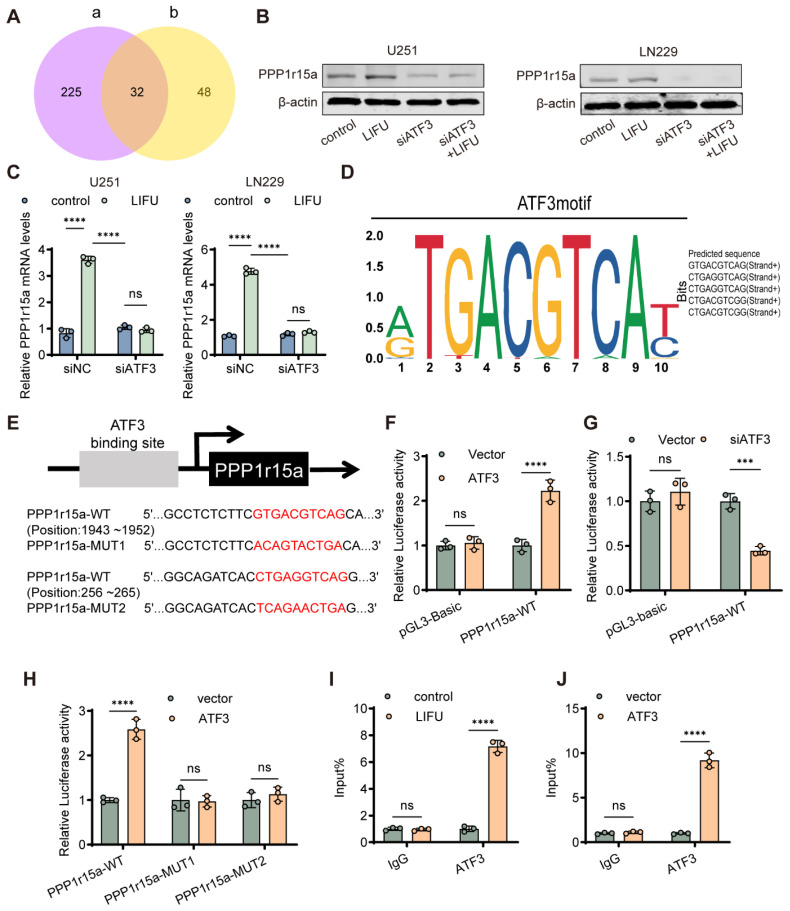
ATF3 binds to the PPP1r15a gene promoter. (**A**) Venn diagram. (**B**) The expression levels of PPP1r15a were determined by Western blot. (**C**) The mRNA expression levels of PPP1r15a were determined. (**D**) JASPAR predicted ATF3-binding sites in the promoter region of PPP1r15a (https://jaspar.elixir.no/ (accessed on 19 December 2024)). (**E**) The sequences of WT/mut PPP1r15a binding sites are indicated (The red text in the figure indicates the alignment of the sequences before and after the mutation). (**F**) ATF3 plasmid was co-transfected with the full-length PPP1r15a promoter in 293T cells. Dual-luciferase reporter assay was performed to measure the activity of the PPP1r15a promoter 48 h after transfection. (**G**) Full-length PPP1r15a promoter plasmid was transfected into the U251 siATF3 cell line. Dual-luciferase reporter assay was performed 48 h after transfection. (**H**) Luciferase activity assays were performed in the indicated cells transfected with PPP1r15a-WT or PPP1r15a-mut promoter-reporter plasmids. (**I**,**J**) ATF3 antibody was used to perform the CHIP-qPCR. The data are presented as the means ± SEM from at least three independent biological replicates. *** *p* < 0.001; **** *p* < 0.0001. WT, wild-type; mut, mutant. The original Western blot figures can be found in Supplementary File S1.

Analysis of the UALCAN database identified ATF3-interacting proteins (Figure S7A), which were cross-referenced with the present sequencing data, yielding 32 overlapping genes (Figure 6A). The STRING database revealed a direct association between ATF3 and PPP1r15a (Figure S7B), consistent with the present sequencing findings (Figure S7C). GeneMANIA further indicated that ATF3 and PPP1r15a are co-involved in ERS response pathways (Figure S7D). The UALCAN analysis indicated a strong positive correlation between ATF3 and PPP1r15a expression (Pearson r = 0.70; Figure S7F). As a key mediator of ERS, PPP1r15a (encoding GADD34) exacerbates ERS when overexpressed. To determine its role in LIFU-induced apoptosis, it was confirmed that PPP1r15a expression was upregulated following LIFU stimulation, along with ATF3, ATF4 and CHOP. Experimental validation revealed that ATF3 knockdown reduced PPP1r15a expression at protein and mRNA levels (Figure 6B,C and Figure S7E). Previous studies have shown that ATF3 directly binds to the promoter region of genes through recognizing nucleotide sequence motifs [14,28]. Therefore, the present study determined whether ATF3 directly binds to the PPP1r15a promoter by recognizing nucleotide sequence motifs. The potential ATF3 binding motifs of the PPP1r15a promoter were predicted by the Jaspar software (2024; Release 10) in the +256 to +1952 of the PPP1r15a transcription start site. The two highest scores of binding motifs were found: (GTGACGTCAG, base position +1943 to +1952) and (CTGAGGTCAG, base position +256 to +265; Figure 6D,E). The effect of ATF3 on the transcriptional activity of the PPP1r15a promoter was investigated by co-transfecting the ATF3 plasmid and full-length PPP1r15a promoter plasmid. The dual-luciferase reporter assay results indicated that ATF3 enhanced the transcriptional activity of the PPP1r15a promoter (Figure 6F). However, when the full-length PPP1r15a promoter plasmid was transfected into U251 siATF3 cells, the transcriptional activity of the PPP1r15a promoter was downregulated in the U251 siATF3 cell line (Figure 6G). The binding sites were mutated to establish the mutant promoter; the mutation of either binding motif (MUT1 or MUT2) significantly reduced ATF3-induced activation (Figure 6H). ChIP-qPCR assays were performed using an anti-ATF3 antibody or control rabbit IgG, and the enrichment of the PPP1r15a promoter region was calculated as a percentage of the input chromatin. LIFU treatment significantly increased the occupancy of ATF3 at the PPP1r15a promoter compared with the IgG group. Similarly, ATF3 overexpression led to a marked increase in promoter enrichment (Figure 6I,J). ATF3-mediated PPP1r15a transcription promotes growth arrest and DNA damage-inducible protein (GADD34) expression. GADD34 dephosphorylates eIF2α through PERK signaling, thereby alleviating translational repression and increasing pro-apoptotic protein synthesis. Next, we performed rescue experiments with ATF3 overexpression following Piezo1 inhibition. Flow cytometry results showed that siPiezo1 significantly attenuated LIFU-induced apoptosis. Notably, when ATF3 was simultaneously overexpressed in the siPiezo1 background (siPiezo1 + ATF3OE), the apoptosis rate was markedly upregulated compared to the LIFU + siPiezo1 group (Figure S8A,B). Furthermore, qPCR analysis of PPP1r15a revealed that the LIFU-induced upregulation of PPP1r15a was substantially suppressed by siPiezo1, and this suppression was effectively restored by ATF3 overexpression in the siPiezo1 cells (Figure S8C). These results further confirm that ATF3 regulates PPP1r15a transcription downstream of Piezo1.

### 3.6. In Vivo Investigation of LIFU for GBM Treatment

**Figure 7:**
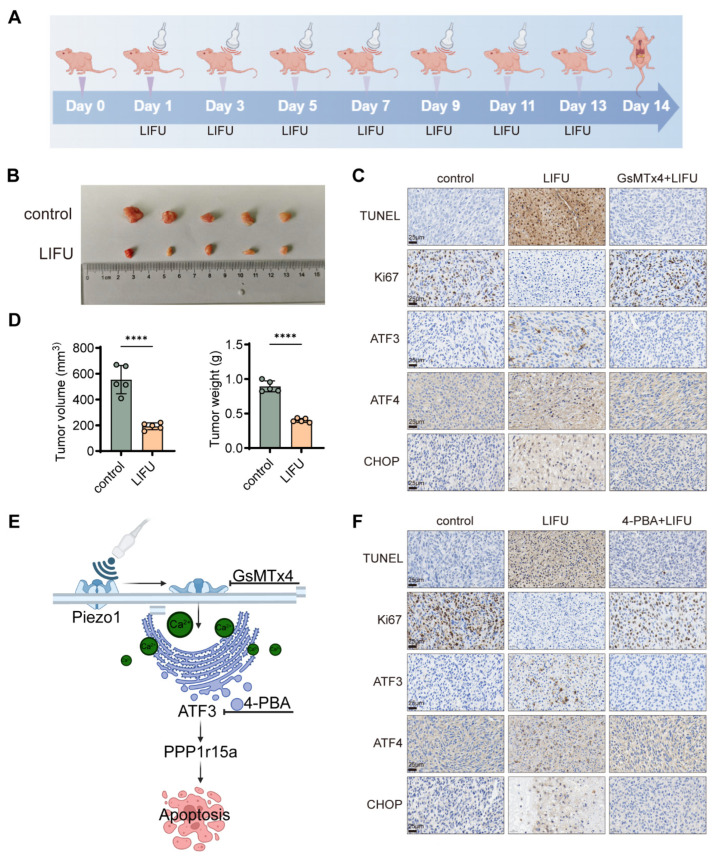
In vivo investigation of LIFU for GBM treatment. (**A**) LIFU intervention diagram. (**B**) Tumor tissue. (**C**) TUNEL, Ki67, ATF3, ATF4 and CHOP expression levels were determined by immunohistochemistry after GsMTx4. (**D**) Tumor volume and tumor weight. (**E**) Mechanism diagram (Created in https://BioRender.com). (**F**) TUNEL, Ki67, ATF3, ATF4 and CHOP expression levels were determined by immunohistochemistry after 4-PBA. The data are presented as the means ± SEM from at least three independent biological replicates. Statistical comparisons among multiple groups were performed using two-way ANOVA followed by Šídák’s multiple comparisons test. **** *p* < 0.0001. LIFU, low-intensity focused ultrasound; GBM, glioblastoma.

To verify the therapeutic effect of LIFU on GBM, a xenograft model was conducted using BALB/c nude mice transplanted with tumor-bearing U251 nude mice. Once every other day, tumors were exposed to LIFU at an intensity of 1 W/cm^2^, 3 min, for a total of seven sessions (Figure 7A). Consistent with prior research [29], our 3 min duration was chosen based on the need to ensure adequate energy delivery to subcutaneous tumors given the attenuation through mouse skin and tissue. The maximum tumor volume in the control group reached 669 mm^3^, with a maximum diameter of 1.4 cm. The tumor volume and weight of the LIFU treatment group were reduced compared with those of the control group on day 7 and day 14 (Figure 7B,D and Figure S8D). Moreover, the TUNEL staining experiment demonstrated that LIFU treatment increased the apoptosis rate of tumor cells. The Ki67 staining experiment demonstrated that LIFU treatment increased the anti-proliferative effects of tumor cells. IHC also indicated that LIFU treatment promoted the expression levels of ATF3, CHOP and ATF4 (Figure 7C,F). The expression levels of ATF3, CHOP and ATF4 were slightly decreased following the application of GsMTx4 or 4-PBA (Figure 7C,F). These findings indicate that LIFU achieves anti-GBM function in vivo, and the underlying mechanism may be correlated with the ERS effect (Figure 7E).

## 4. Discussion

GBM is the most common primary neurological tumor, characterized by a high degree of malignancy and a five-year survival rate of only 10% [30]. Despite advances in oncology, treatment strategies for GBM have shown little improvement over the past 40 years, with surgery and chemotherapy remaining the standard treatments of care [31]. Therefore, there is an urgent need to explore novel therapeutic strategies for treating GBM. LIFU can generate mechanical forces that target tumor tissues without damaging normal tissues [32] and has been indicated to have antitumor effects [29]. However, the specific mechanism of LIFU against GBM remains unclear. In the present study, the effects and molecular mechanisms of LIFU on the growth and progression of GBM were preliminarily explored. The findings revealed an association between ERS and LIFU-induced apoptosis, and the mechanisms behind LIFU-induced ERS were revealed from the perspective of mechanosensitive ion channels. It was proposed that the mechanical force generated by LIFU targets the apoptosis of tumor cells, with the potential mechanism being that LIFU activates the mechanosensitive channel on the cell surface, Piezo1, when acting on cells. Among them, the transcription factor ATF3 binds to the PPP1r15a promoter as the primary ERS factor, promoting the transcription of PPP1r15a, thereby exacerbating the ERS response and inducing apoptosis of tumor cells [33].

Tumor cells exhibit greater sensitivity than normal cells to mechanical stimuli, making them more likely to be destroyed under the same mechanical force [34]. Previous studies have demonstrated the importance of mechanomapoptosis (mechanically induced apoptosis). For instance, Tijore et al. [35] observed that physiologically relevant periodic mechanical stretching inhibits the growth of transformed tumor cells and activates apoptosis. Similarly, mechanical forces such as shear or ultrasound [36,37] can induce mechanical apoptosis in tumor cells. This study demonstrated that LIFU activates the mitochondrial apoptotic pathway through the induction of calcium influx. Shear forces suppress cancer through Smad 1/5 and p38 MAPK [38], while the combination of fluid shear stress and TRAIL induces tumor apoptosis through caspase activation [39,40]. Due to the additional advantages of ultrasound, such as non-invasiveness and the ability to penetrate deep tissues, ultrasound has been widely explored for the development of tumor mechanological treatments. Among them, HIFU therapy primarily utilizes its thermal effect, which non-selectively ablates tissue, destroying normal cells around tumors [41]. By contrast, LIFU has been found to achieve targeted killing of tumor cells through non-thermomechanical effects, including stretching, compression and shear stress [6]. Previous studies on LIFU targeting tumor cells have indicated that LIFU induces apoptosis in the human hepatocellular carcinoma cells SMMC-7721 in vitro [8], and LIFU can rapidly inhibit tumor growth without damaging healthy tissues [29]. While this study established a relationship between LIFU and apoptosis, it did not investigate the mechanism of calcium influx nor the subsequent process by which this influx initiates apoptosis. Ultrasound-mediated mechanical forces cause selective killing of the tumor, without notable damage to normal cells, and the effect of ultrasound-specific damage to tumor tissue is more notable over time [36]. Consequently, LIFU-based mechanical force therapy is worthy of further development.

Through transcriptome sequencing, GO, KEGG and GSEA, it was found that differential genes were significantly enriched in ERS, cell cycle arrest and apoptosis pathways. This suggests that LIFU can promote apoptosis in GBM cells, consistent with previous studies [29,42]. A study has indicated that LIFU can induce ERS in endothelial cells [43], which is similar to the results of the present study, prompting further investigation of the effect of LIFU on ERS in GBM cells. Subsequently, key molecules were detected by PCR, and the results indicated that the mRNA levels of ATF3, XBP1, CHOP, ATF4 and PPP1r15a were significantly increased in the LIFU intervention group. Based on this, it was concluded that when external stimuli stimulate cells, they activate the UPR effect, which in turn activates ERS.

The most notable gene in the ERS pathway is ATF3, which has been indicated to have anti-cancer properties [44]. Following LIFU intervention in ATF3 knockout GBM cells, the levels of ATF4, PPP1r15a and CHOP were lower than those in the LIFU group alone, proving that ATF3 is a key molecule for LIFU to treat GBM. The results of the JASPAR database and transcriptome sequencing were obtained from PPP1r15a, which is closely associated with ATF3. The findings were obtained by ChIP experiments, and ATF3 was bound to the promoter region of PPP1r15a as a transcription factor to initiate the transcription of PPP1r15a. PPP1r15a is a pro-apoptotic modulator that is crucial in inducing apoptosis in tumor cells [45]. Studies have validated that targeting ERS is a promising tumor treatment strategy [46], such as phenylalanine deprivation and inhibition of NRF2 [14,16]. Similar to these findings, the present study indicates that ATF3 is activated in LIFU treatment, thereby activating its downstream ERS-related signaling pathway, which ultimately promotes apoptosis of GBM cells and exerts antitumor effects. In the present study, PPP1r15a was confirmed to be a downstream protein regulated by ATF3 in GBM, and LIFU targeted the ATF3/PPP1r15a pathway with pronounced anti-GBM effects.

Previous studies have indicated that ultrasound induces calcium ion entry through the mechanically sensitive Piezo 1 channel on the surface of breast cancer cells, which destroys microtubules through calpain activation [37]. Piezo1 is highly expressed in pancreatic ductal adenocarcinoma (PDAC) cells and is activated by ultrasound microvesicles, which are closely associated with apoptosis in PDAC cell lines and tumors [47]. Tumor cells undergo apoptosis through the calpainin-dependent mitochondrial pathway, which depends on calcium entry through the mechanically sensitive Piezo1 channel [36]. Given its mechanosensitive nature, we hypothesized that external mechanical stimulation via LIFU could modulate Piezo1 activity and trigger downstream cellular responses, including ERS. In the present study, mRNA and protein expression levels of Piezo1 were significantly increased following LIFU intervention in GBM cells. When Piezo1 is opened, there is a large influx of calcium ions, and calcium ion overload can disrupt the homeostasis of the ER, causing post-ER cell activation compensation mechanisms, such as UPR [48]. UPR can relieve cellular stress by repairing misfolded proteins in the nucleus and cytoplasm. However, irreversible ERS can lead to cell death [49]. GsMTx4 or siPiezo1 inhibited Piezo1, followed by LIFU, and it was found that ERS were reduced, which demonstrated that Piezo1 plays a crucial role in GBM treatment in LIFU-induced ERS. In this study, Fluo-4 AM staining was used to monitor intracellular Ca^2+^ changes, and the average fluorescence intensity was quantified to provide semi-quantitative evidence of Ca^2+^ influx. However, we did not examine the magnitude or kinetics of the Ca^2+^ transients, nor did we distinguish between extracellular Ca^2+^ influx and ER Ca^2+^ release. Future experiments using Ca^2+^-free buffers, EGTA, and ER Ca^2+^ channel inhibitors (e.g., 2-APB) are required to fully resolve the origin and dynamics of the Ca^2+^ signal induced by LIFU. Another limitation of this study is that GsMTx4 is not a specific inhibitor of Piezo1. At the concentrations used in our experiments, it may also inhibit Piezo2 and other mechanosensitive channels [50]. Future studies should therefore employ more selective Piezo1 antagonists to further validate the specificity of the observed effects. A further limitation of the present study is the use of subcutaneous xenograft models, which do not fully recapitulate the intracranial tumor microenvironment, the blood–brain barrier (BBB) or the native biomechanical context of GBM. Future work must systematically investigate the biological impact of LIFU-induced mechanical forces using orthotopic and patient-derived xenograft models.

## 5. Conclusions

In conclusion, ultrasound-mediated mechanical forces can induce apoptosis in tumor cells across various tissues and microenvironments, suggesting a safe, non-invasive approach to enhance tumor treatment. The present study highlighted the importance of LIFU in GBM treatment through its killing effect on GBM cells by mechanical force. The present findings reveal a new anti-cancer effect of LIFU and its biological mechanisms, offering insights for further exploration of treatment strategies for GBM.

## Data Availability

All data generated or analyzed during this study are included in this published article. [geo] GEO Submission (GSE314685) [NCBI tracking system #25499988]. link: https://www.ncbi.nlm.nih.gov/geo/query/acc.cgi?acc=GSE314685 (accessed on 25 December 2025).

## Supplementary Materials

The following supporting information can be downloaded at: https://www.mdpi.com/article/10.3390/cancers18091445/s1, File S1: The original Western blot figures; Figure S1: LIFU exerts potent antitumor effects. (A) Acoustic pressure variations propagating along the Z-axis of LIFU. (B) Variation of acoustic pressure over time at the same location. (C) Temperature at the LIFU focal point were monitored in control using a FLIR thermal imaging camera. (D) Temperature at the LIFU focal point were monitored in LIFU using a FLIR thermal imaging camera. (E) Cell viability of U251 and LN229 cells treated with LIFU. (F) Cell counting and viability detection after Trypan Blue staining. (G) The corresponding statistical results of the colony formation rate. (H) The corresponding statistical results of apoptosis rate. (I) Cell viability of HT22 cells treated with LIFU. (J) The corresponding statistical results of the relative protein expression values. (K) The corresponding statistical results of the relative protein expression values. The data are presented as the means ± SEM from at least three independent biological replicates (* *p* <0.05; ** *p* < 0.01; *** *p* < 0.001; **** *p* < 0.0001; *n* = 3). LIFU, low-intensity focused ultrasound; Figure S2: Piezo1 has been shown to mediate the ERS effect. (A) ATF4, CHOP and ATF3 expression were determined by western blotting after LIFU. (B) The corresponding statistical results of the relative protein expression values. (C) Representative images of EdU in U251 and LN229 cells with different treatments. (D) The corresponding statistical results of the EdU-positive percentage. (E,F) The corresponding statistical results of the relative protein expression values. The data are presented as the means ± SEM from at least three independent biological replicates (* *p* < 0.05; ** *p* < 0.01; *** *p* < 0.001; **** *p* < 0.0001; *n* = 3); Figure S3: siPiezo1 alleviated LIFU-induced ERS. (A) The corresponding statistical results of the mean fluorescence intensity values. (B) Relative Piezo1 mRNA levels of siPiezo1. (C) Representative IF images of Piezo1 and siPiezo1. (D) ATF3 expression were determined by western blotting after LIFU and siPiezo1. (E) The corresponding statistical results of the relative protein expression values. (F) Annexin-V/PI bound U251 or LN229 cells were counted. (G) ATF3 expression were determined by western blotting after Yoda1. The data are presented as the means ± SEM from at least three independent biological replicates (* *p* < 0.05; ** *p* < 0.01; *** *p* < 0.001; **** *p* < 0.0001; *n* = 3); Figure S4: ATF3 was important in LIFU-induced apoptosis. (A) Intracellular calcium levels were labeled by Fluo-4 (green) after different interventions. (B) The corresponding statistical results of the mean fluorescence intensity values.(C) The corresponding statistical results of the ATF3 protein expression values. (D) The corresponding statistical results of the EdU-positive percentage. (E) The corresponding statistical results of the cell cycle in U251. (F) The corresponding statistical results of the cell cycle in LN229. The data are presented as the means ± SEM from at least three independent biological replicates (* *p* < 0.05; ** *p* < 0.01; *** *p* < 0.001; **** *p* < 0.0001; *n* = 3). IF, immunofluorescent; Figure S5: siATF3 alleviated LIFU-induced apoptosis and ERS. (A) The corresponding statistical results of the apoptosis protein expression values in U251. (B) The corresponding statistical results of the apoptosis protein expression values in LN229. (C) The corresponding statistical results of the ERS protein expression values in U251. (D) The corresponding statistical results of the ERS protein expression values in LN229. The data are presented as the means ± SEM from at least three independent biological replicates (* *p* < 0.05; ** *p* < 0.01; *** *p* < 0.001; **** *p* < 0.0001; *n* = 3); Figure S6: 4-PBA suppressed the ERS induced by LIFU. (A) Colony formation assays were performed with different treatments. (B) The corresponding statistical results of the colony formation. (C) Annexin-V/PI bound U251 or LN229 cells were counted. (D) The corresponding statistical re-sults of apoptosis rate. (E) EIF2α and p-EIF2α expression were determined by western blotting after LIFU and 4-PBA intervention. (F) The corresponding statistical results of the ERS protein expression values. The data are presented as the means ± SEM from at least three independent biological replicates (** *p* < 0.01; *** *p* < 0.001; **** *p* < 0.0001; *n* = 3); Figure S7: The relationship between ATF3 and PPP1r15a was analyzed by bioinformatics. (A) UALCAN analysis (https://ualcan.path.uab.edu/index.html). (B) String data (https://string-db.org/). (C) Cytoscape analysis. (D) GeneMANIA (https://genemania.org/). (E) The corresponding statistical results of the relative protein expression values. (F) UALCAN analysis (https://ualcan.path.uab.edu/index.html). The data are presented as the means ± SEM from at least three independent biological replicates (* *p* < 0.05; **** *p* < 0.0001; *n* = 3); Figure S8: Rescue experiment with Piezo1 knockdown and ATF3 overexpression. (A) Annexin-V/PI bound U251 or LN229 cells were counted. (B) The corresponding statistical re-sults of apoptosis rate. (C) The mRNA expression levels of PPP1r15a were determined. (D) Individual growth curves of tumors in the control and LIFU groups. The data are presented as the means ± SEM from at least three independent biological replicates (* *p* < 0.05; ** *p* < 0.01; *** *p* < 0.001; **** *p* < 0.0001; *n* = 3); Table S1: Primers for randomly selected DEGs used for q-PCR analysis; Table S2: PPP1r15a mutant sequence; Table S3: Primers used for siRNA analysis.
