# Supplementary material for: Piezo1-ATF3-PPP1r15a Axis Transduces Mechanical Stress into Apoptosis in Glioma Under Low-Intensity Focused Ultrasound
Source: Cancers (Basel). 2026 Apr 30;18(9):1445. doi: 10.3390/cancers18091445 (PMC13162950; doi:10.3390/cancers18091445)
Supplement: Supplementary file 1 [file cancers-18-01445-s001.zip › cancers-4240230-supplementary Figures.pdf]

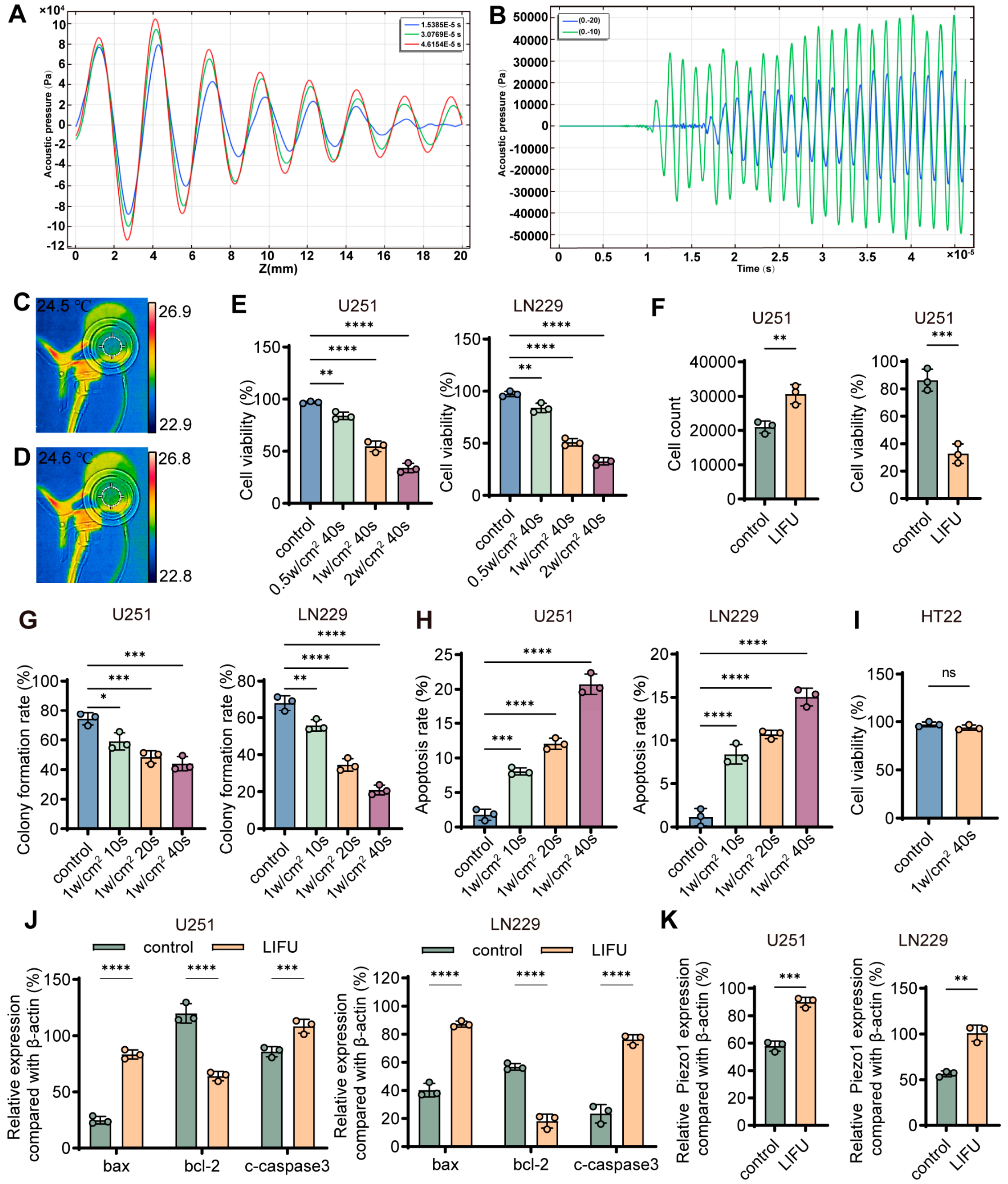


**Figure S1.** LIFU exerts potent antitumor effects. (A) Acoustic pressure variations propagating along the Z-axis of LIFU. (B) Variation of acoustic pressure over time at the same location. (C) Temperature at the LIFU focal point were monitored in control using a FLIR thermal imaging camera. (D) Temperature at the LIFU focal point were monitored in LIFU using a FLIR thermal imaging camera. (E) Cell viability of U251 and LN229 cells treated with LIFU. (F) Cell counting and viability detection after Trypan Blue staining. (G) The corresponding statistical results of the colony formation rate. (H) The corresponding statistical results of apoptosis rate. (I) Cell

viability of HT22 cells treated with LIFU. (J) The corresponding statistical results of the relative protein expression values. (K) The corresponding statistical results of the relative protein expression values. The data are presented as the means ± SEM from at least three independent biological replicates ($^{*}p < 0.05$; $^{**}p < 0.01$; $^{***}p < 0.001$; $^{****}p < 0.0001$; n = 3). LIFU, low-intensity focused ultrasound.

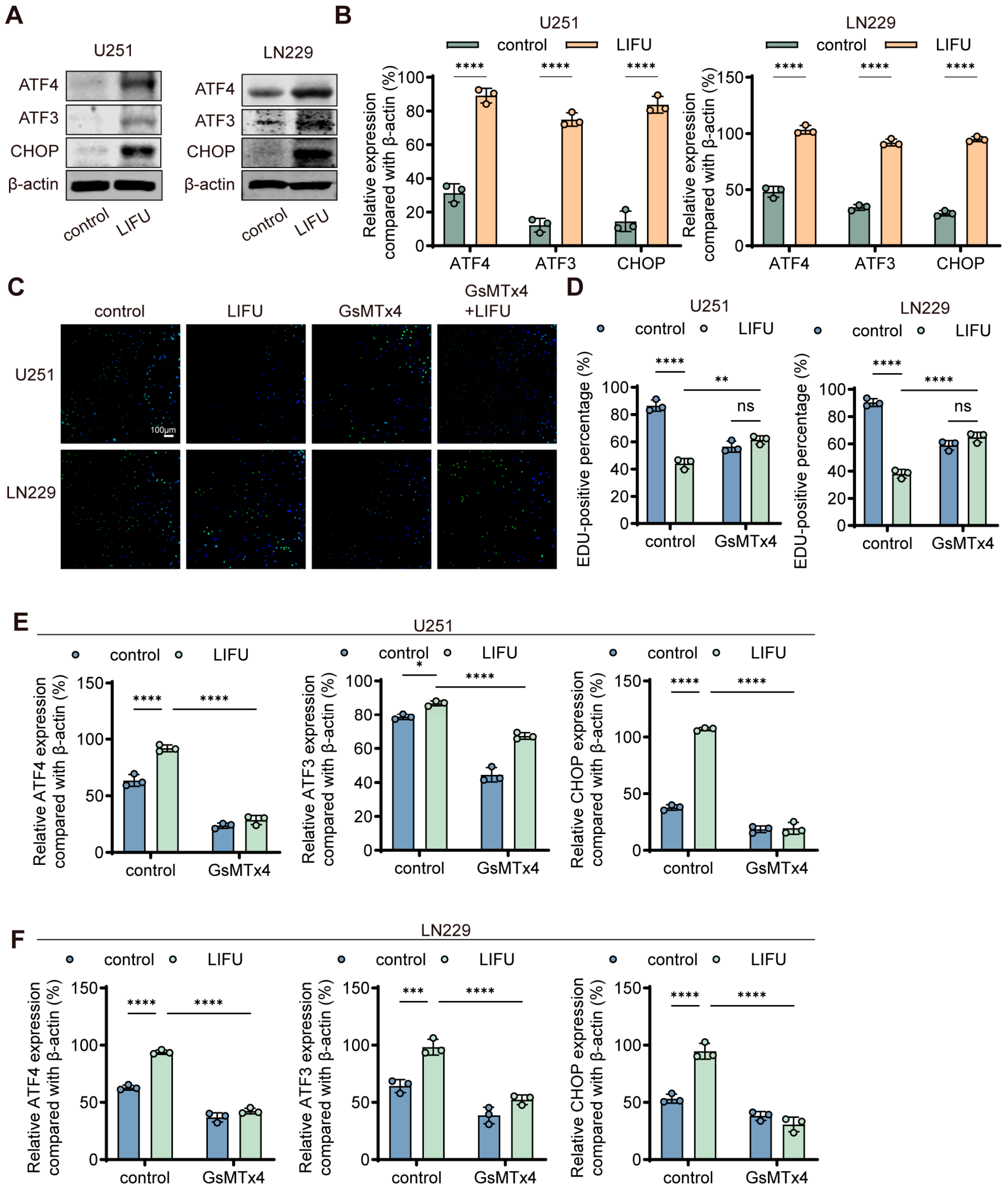


**Figure S2.** Piezo1 has been shown to mediate the ERS effect. (A) ATF4, CHOP and ATF3 expression were determined by western blotting after LIFU. (B) The corresponding statistical results of the relative protein expression values. (C) Representative images of EdU in U251 and LN229 cells with different treatments. (D) The corresponding statistical results of the EdU-positive percentage. (E,F) The corresponding statistical results of the relative protein expression values. The data are presented as the means ± SEM from at least three independent biological replicates ($^{*}p < 0.05$; $^{**}p < 0.01$; $^{***}p < 0.001$; $^{****}p < 0.0001$; n = 3).

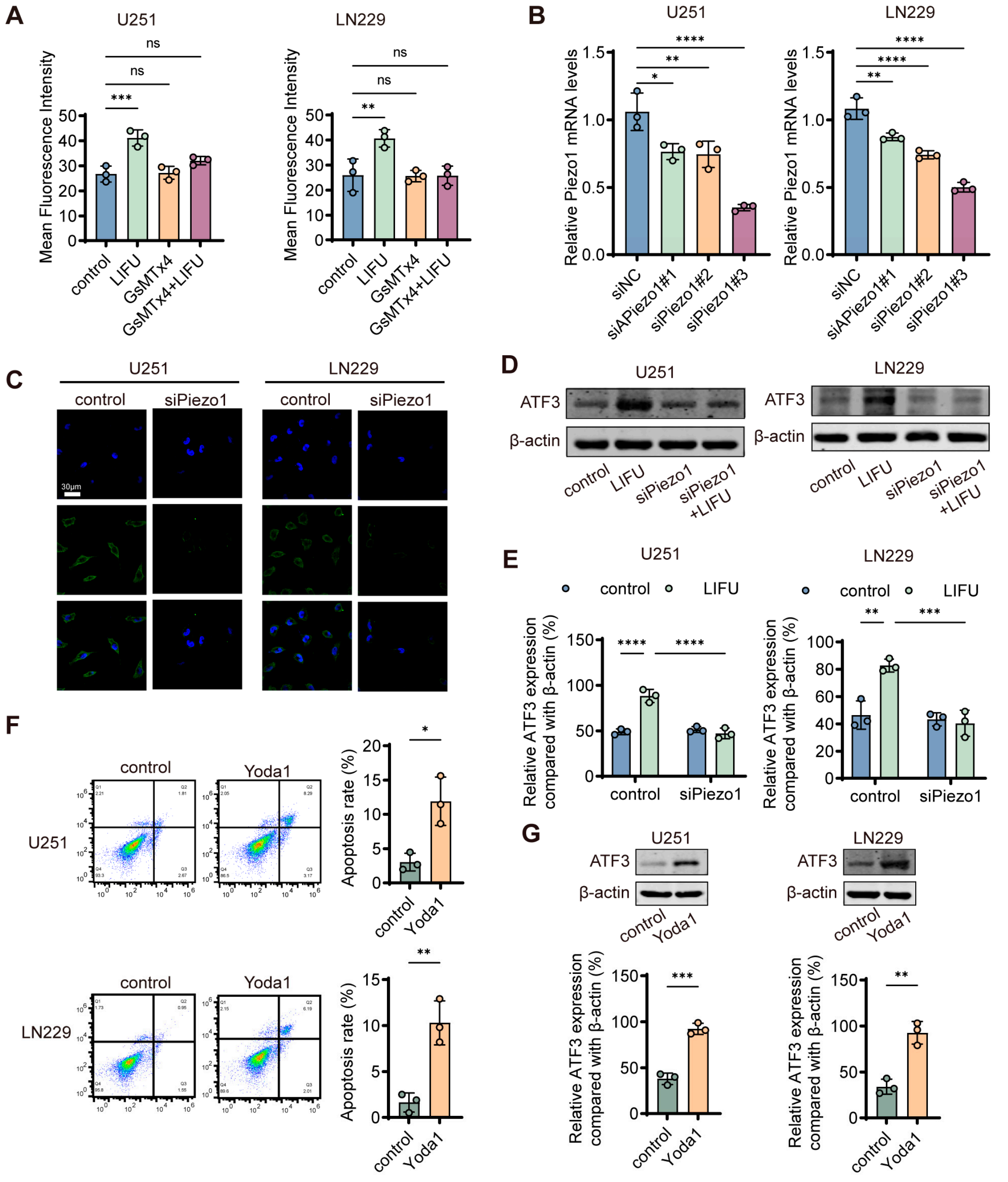


**Figure S3.** siPiezo1 alleviated LIFU-induced ERS. (A) The corresponding statistical results of the mean fluorescence intensity values. (B) Relative Piezo1 mRNA levels of siPiezo1. (C) Representative IF images of Piezo1 and siPiezo1. (D) ATF3 expression were determined by western blotting after LIFU and siPiezo1. (E) The corresponding statistical results of the relative protein expression values. (F) Annexin-V/PI bound U251 or LN229 cells were counted. (G) ATF3 expression were determined by western blotting after Yoda1. The data are presented as the means ± SEM from at least three

independent biological replicates ($^{*}p < 0.05$; $^{**}p < 0.01$; $^{***}p < 0.001$; $^{****}p < 0.0001$; n = 3).

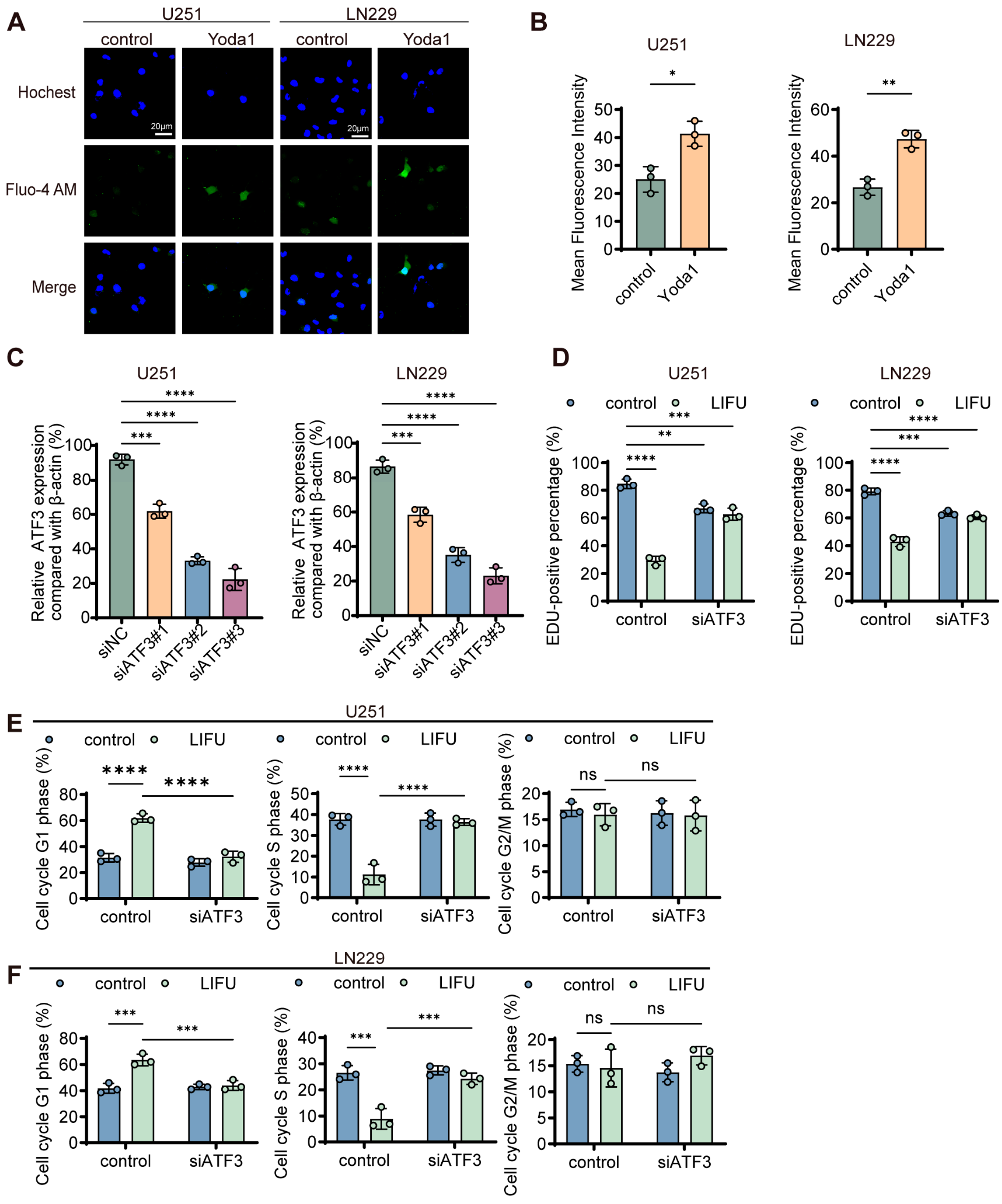


**Figure S4.** ATF3 was important in LIFU-induced apoptosis. (A) Intracellular calcium levels were labeled by Fluo-4 (green) after different interventions. (B) The corresponding statistical results of the mean fluorescence intensity values.(C) The corresponding statistical results of the ATF3 protein expression values. (D) The corresponding statistical results of the EdU-positive percentage. (E) The corresponding statistical results of the cell cycle in U251. (F) The corresponding

statistical results of the cell cycle in LN229. The data are presented as the means ± SEM from at least three independent biological replicates ($^{*}p < 0.05$; $^{**}p < 0.01$; $^{***}p < 0.001$; $^{****}p < 0.0001$; n = 3). IF, immunofluorescent.

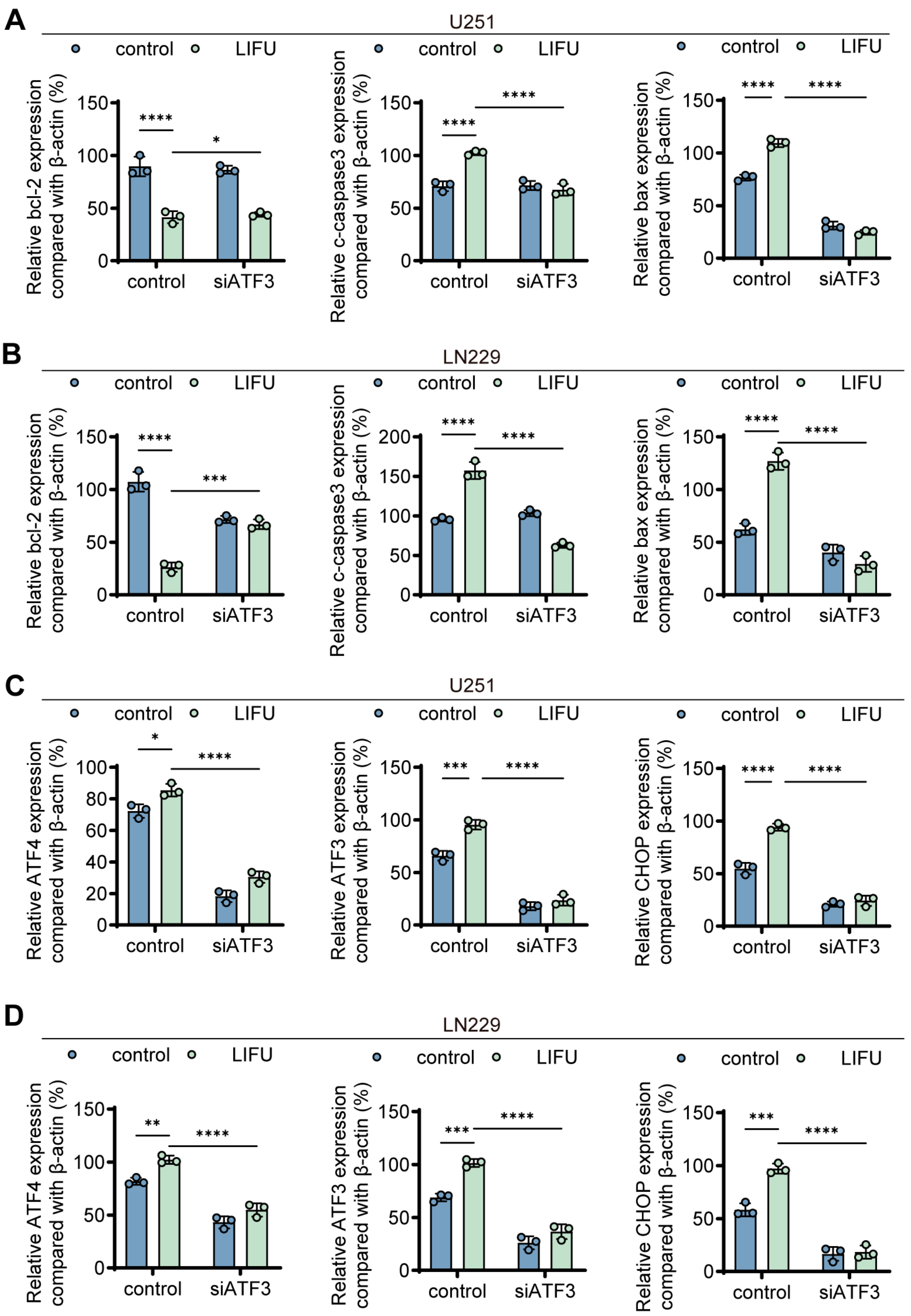


**Figure S5.** siATF3 alleviated LIFU-induced apoptosis and ERS. (A) The corresponding statistical results of the apoptosis protein expression values in U251. (B) The corresponding statistical results of the apoptosis protein expression values in LN229. (C) The corresponding statistical results of the ERS protein expression values in U251. (D) The corresponding statistical results of the ERS protein expression values in LN229. The data are presented as the means ± SEM from at least three

independent biological replicates ($^{*}p < 0.05$; $^{**}p < 0.01$; $^{***}p < 0.001$; $^{****}p < 0.0001$; n = 3).

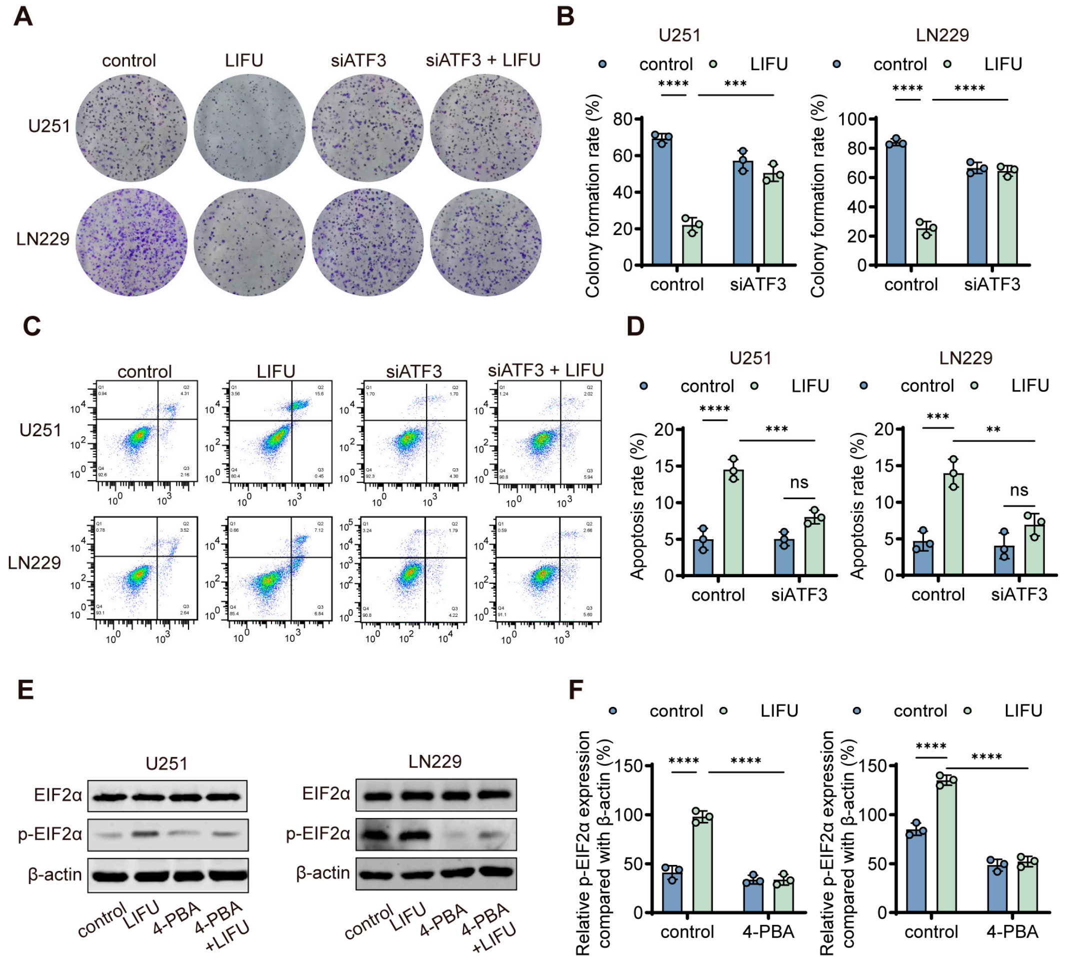


**Figure S6.** 4-PBA suppressed the ERS induced by LIFU. (A) Colony formation assays were performed with different treatments. (B) The corresponding statistical results of the colony formation. (C) Annexin-V/PI bound U251 or LN229 cells were counted. (D) The corresponding statistical re-sults of apoptosis rate. (E) EIF2α and p-EIF2α expression were determined by western blotting after LIFU and 4-PBA intervention. (F) The corresponding statistical results of the ERS protein expression values. The data are presented as the means ± SEM from at least three independent biological replicates ($^{**}p < 0.01$; $^{***}p < 0.001$; $^{****}p < 0.0001$; n = 3).

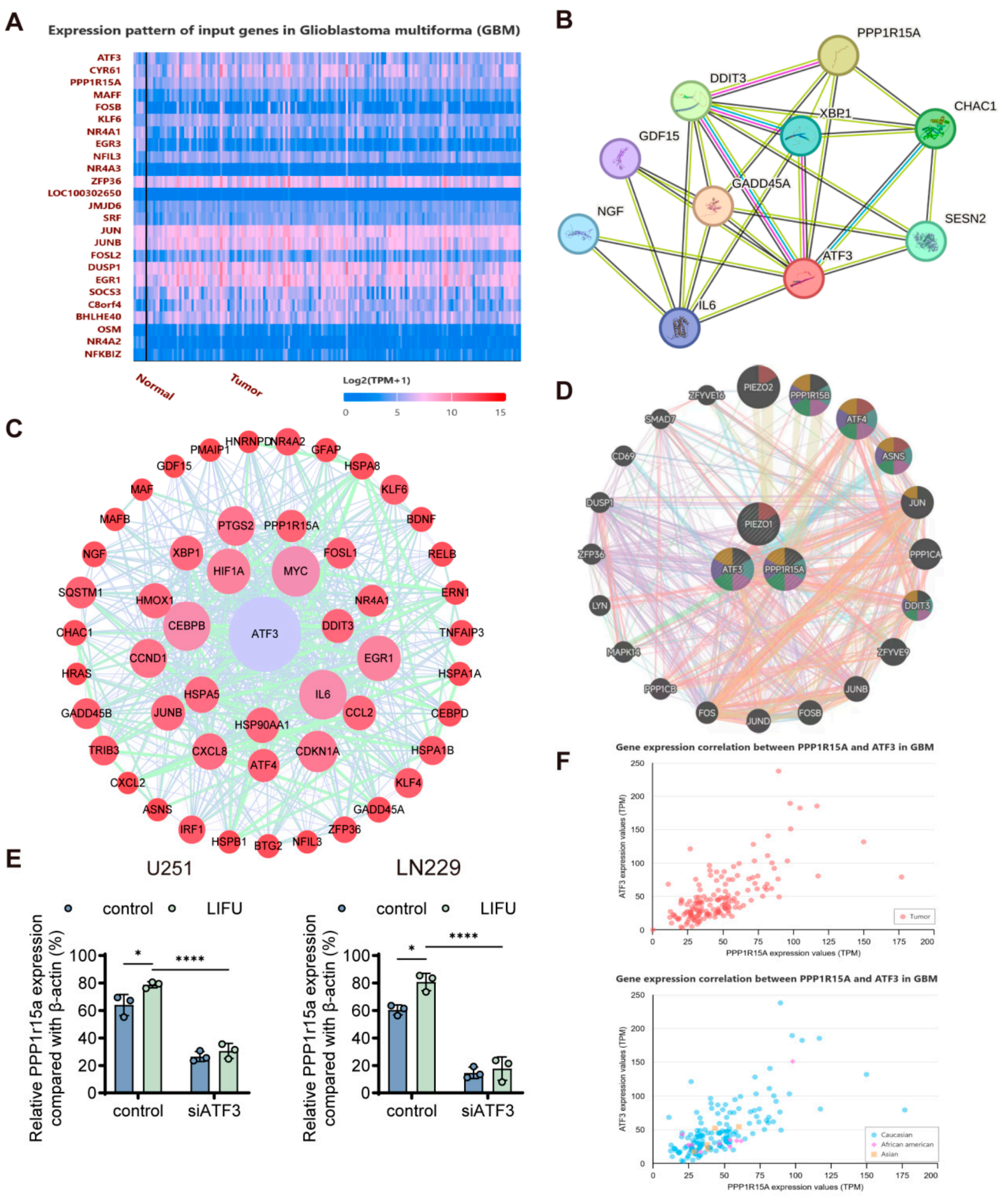


**Figure S7.** The relationship between ATF3 and PPP1r15a was analyzed by bioinformatics. (A) UALCAN analysis (https://ualcan.path.uab.edu/index.html). (B) String data (https://string-db.org/). (C) Cytoscape analysis. (D) GeneMANIA (https://genemania.org/). (E) The corresponding statistical results of the relative protein expression values. (F) UALCAN analysis (https://ualcan.path.uab.edu/index.html). The data are presented as the means ± SEM from at least three independent biological replicates ($^{*}p < 0.05$; $^{****}p < 0.0001$; n = 3).

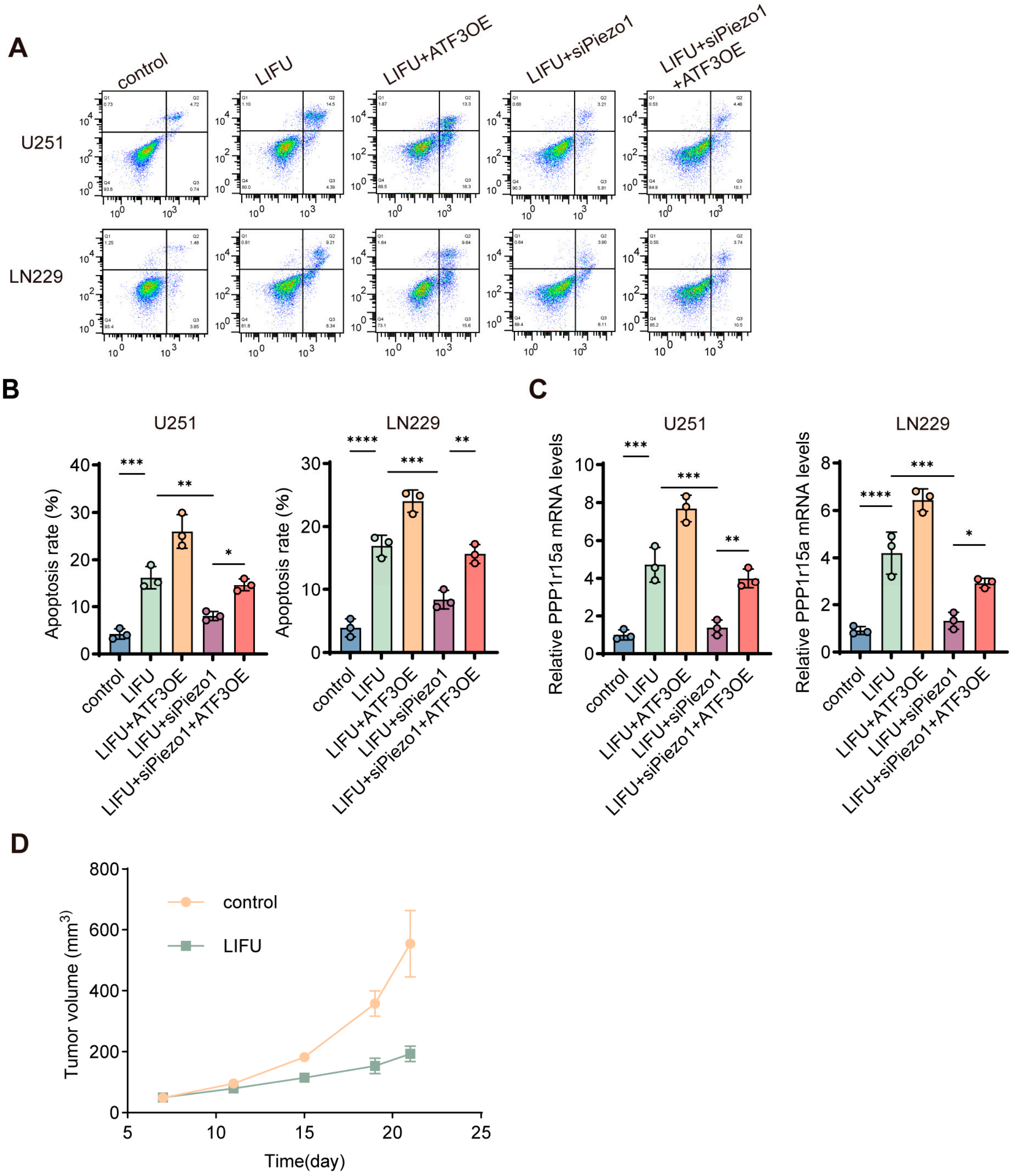


**Figure S8.** Rescue experiment with Piezo1 knockdown and ATF3 overexpression. (A) Annexin-V/PI bound U251 or LN229 cells were counted. (B) The corresponding statistical re-sults of apoptosis rate. (C) The mRNA expression levels of PPP1r15a were determined. (D) Individual growth curves of tumors in the control and LIFU groups. The data are presented as the means ± SEM from at least three independent biological replicates ($^{*}p < 0.05$; $^{**}p < 0.01$; $^{***}p < 0.001$; $^{****}p < 0.0001$; n = 3).
