# Supplementary material for: Piezo1-ATF3-PPP1r15a Axis Transduces Mechanical Stress into Apoptosis in Glioma Under Low-Intensity Focused Ultrasound
Source: Cancers (Basel). 2026 Apr 30;18(9):1445. doi: 10.3390/cancers18091445 (PMC13162950; doi:10.3390/cancers18091445)
Supplement: Supplementary file 1 [file cancers-18-01445-s001.zip › cancers-4240230-supplementary Tables.pdf]

Table S1. Primers for randomly selected DEGs used for q-PCR analysis.

Gene		Sequence
ATF3	Forward	CTGGAATCAGTCACTGTCA
	Reverse	CTTCTCCGACTCTTTCTG
GAPDH	Forward	GTCTCCTCTGACTTCAACAGCG
	Reverse	ACCACCCTGTTGCTGTAGCCAA
PIEZO1	Forward	CCTGGAGAAGACTGACGGCTAC
	Reverse	ATGCTCCTTGGATGGTGAGTCC
CHOP	Forward	GGTATGAGGACCTGCAAGAGGT
	Reverse	CTTGTGACCTCTGCTGGTTCTG
XBP1	Forward	CTGCCAGAGATCGAAAGAAGGC
	Reverse	CTCCTGGTTCTCAACTACAAGGC
ATF4	Forward	TTCTCCAGCGACAAGGCTAAGG
	Reverse	CTCCAACATCCAATCTGTCCCG
PPP1r15a	Forward	TCCGACTGCAAAGGCGGCTCA
	Reverse	CAGCCAGGAAATGGACAGTGAC

Table S2. PPP1r15a mutant sequence.

Gene	Sequence
PPP1r15a-MUT1	GCTAGCGCTGGGGCCTGTGGGCTAAATTGGGGTAGG<br>AGGCCAGGCACAGTGGATCACAACTGTAATTCCAGC<br>ACTTTGGGAAGCCAAGGCGGGCAGATCACTCAGAA<br>CTGAGAGTTCAAGACCAGCCTGGTCGGCCGGGTGCG<br>GTGGCTCACACCTGTAATCCCAGCACTTTGGGAGGC<br>CGAGGCGGGTGGATCACAAGGTCAGGACTCGAG
PPP1r15a-MUT2	GCTAGCGACAGTTCGTTTGTTGGAGGGGCGTGGTCA<br>CGCTCGGAAACTCCGCCGTGACGTTGCAAAAGCTGG<br>AATCTCCGCGAGAAGTCCTGTCTTACTTCCACTTCCC<br>ACCCTTCGGGTTGCGGTCTCGAAACCCCGCCTCTCTT<br>CACAGTACTGACACGCCGGGCGCGGTAGGCTATAAA<br>AGCCTAGTGGCCATTGTGTTCGTTCTCGAG

Table S3. Primers used for siRNA analysis.

Gene		Sequence
siNC	Sense	UUCUCCGAACGUGUCACGUTT
	Antisense	ACGUGACACGUUCGGAGAATT
siATF3#1	Sense	CGAGAAGCAGCAUUUGAUA/dT//dT/
	Antisense	UAUCAAAUGCUGCUUCUCG/dT//dT/
siATF3#2	Sense	GGAAAGUGUGAAUGCUGAA/dT//dT/
	Antisense	UUCAGCAUUCACACUUUCC/dT//dT/
siATF3#3	Sense	GAAACAAGAAGAAGGAGAA/dT//dT/
	Antisense	UUCUCCUUCUUCUUGUUUC/dT//dT/
siPiezo1#1	Sense	CCCACGCGCUGGUCCUCAA/dT//dT/
	Antisense	UUGAGGACCAGCGCGUGGG/dT//dT/
siPiezo1#2	Sense	GUGUCUACCUGCUGCUCUU/dT//dT/
	Antisense	AAGAGCAGCAGGUAGACAC/dT//dT/
siPiezo1#3	Sense	CCGACACCGAGGCUGAUAA/dT//dT/
	Antisense	UUAUCAGCCUCGGUGUCGG/dT//dT/
