# Supplementary material for: Piezo1-ATF3-PPP1r15a Axis Transduces Mechanical Stress into Apoptosis in Glioma Under Low-Intensity Focused Ultrasound
Source: Cancers (Basel). 2026 Apr 30;18(9):1445. doi: 10.3390/cancers18091445 (PMC13162950; doi:10.3390/cancers18091445)
Supplement: Supplementary file 1 [file cancers-18-01445-s001.zip › File S1. The original Western blot figures.pdf]

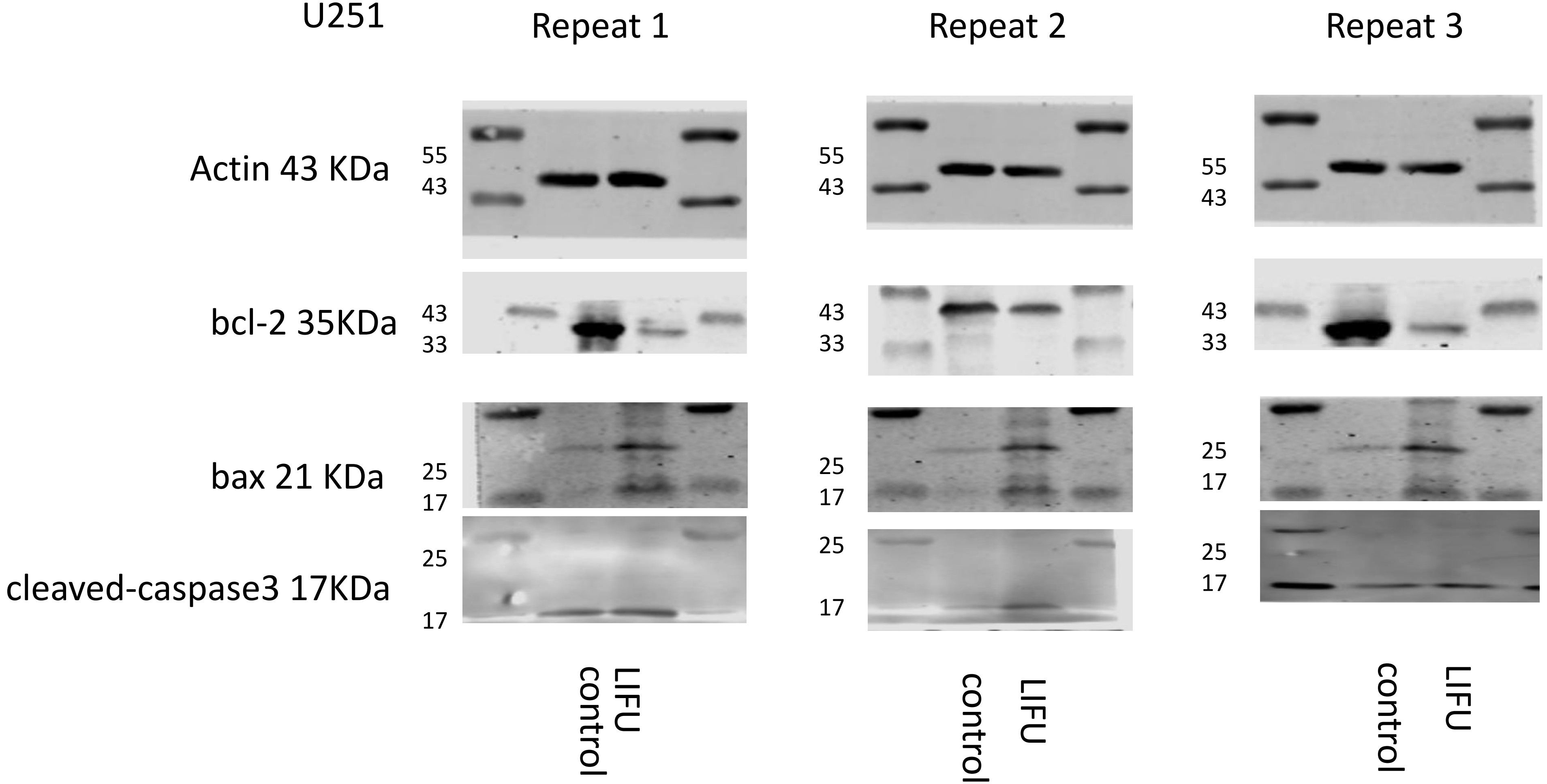

U251
Repeat 1
Repeat 2
Repeat 3
Actin 43 KDa
bcl-2 35KDa
bax 21 KDa
cleaved-caspase3 17KDa
55
43
43
33
25
17
25
17
control
LIFU

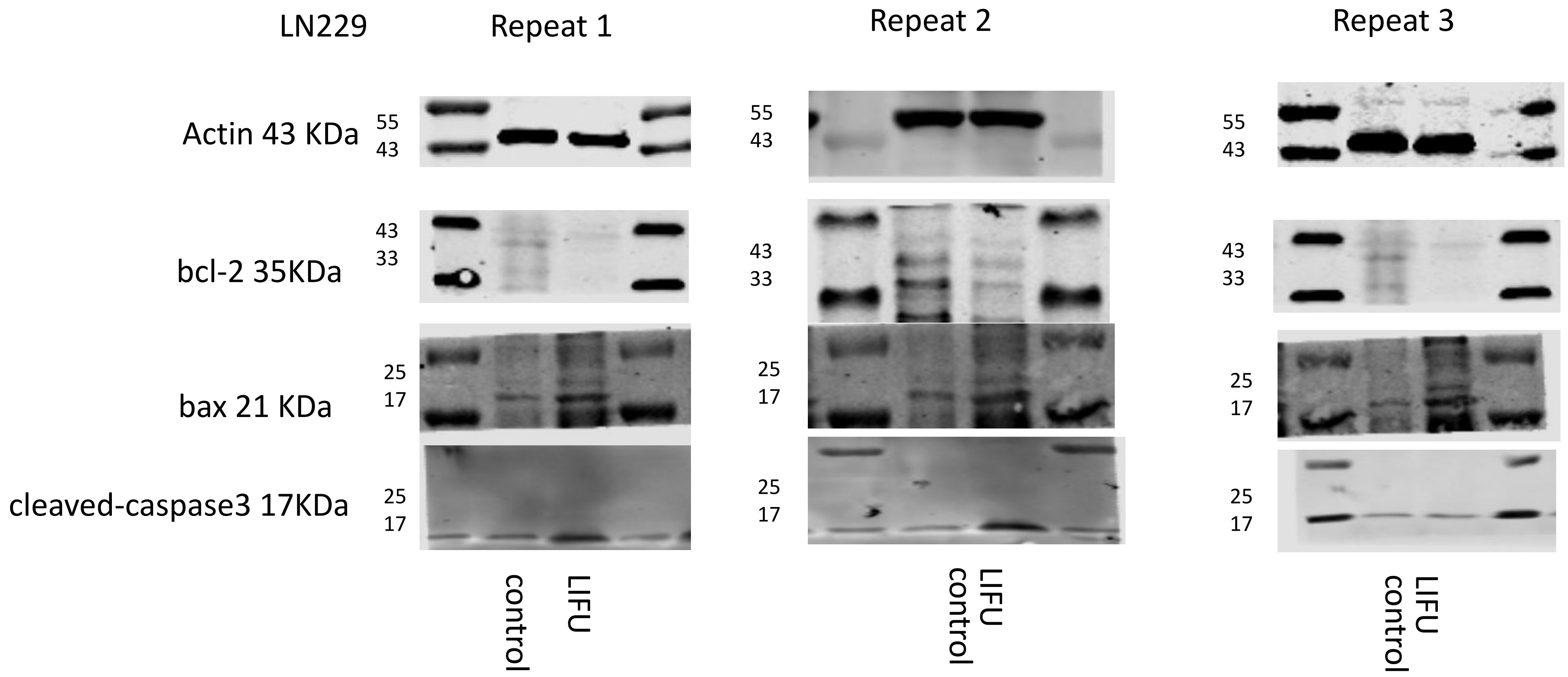

LN229
Repeat 1
Repeat 2
Repeat 3
Actin 43 KDa
bcl-2 35KDa
bax 21 KDa
cleaved-caspase3 17KDa
55
43
33
25
17
control
LIFU

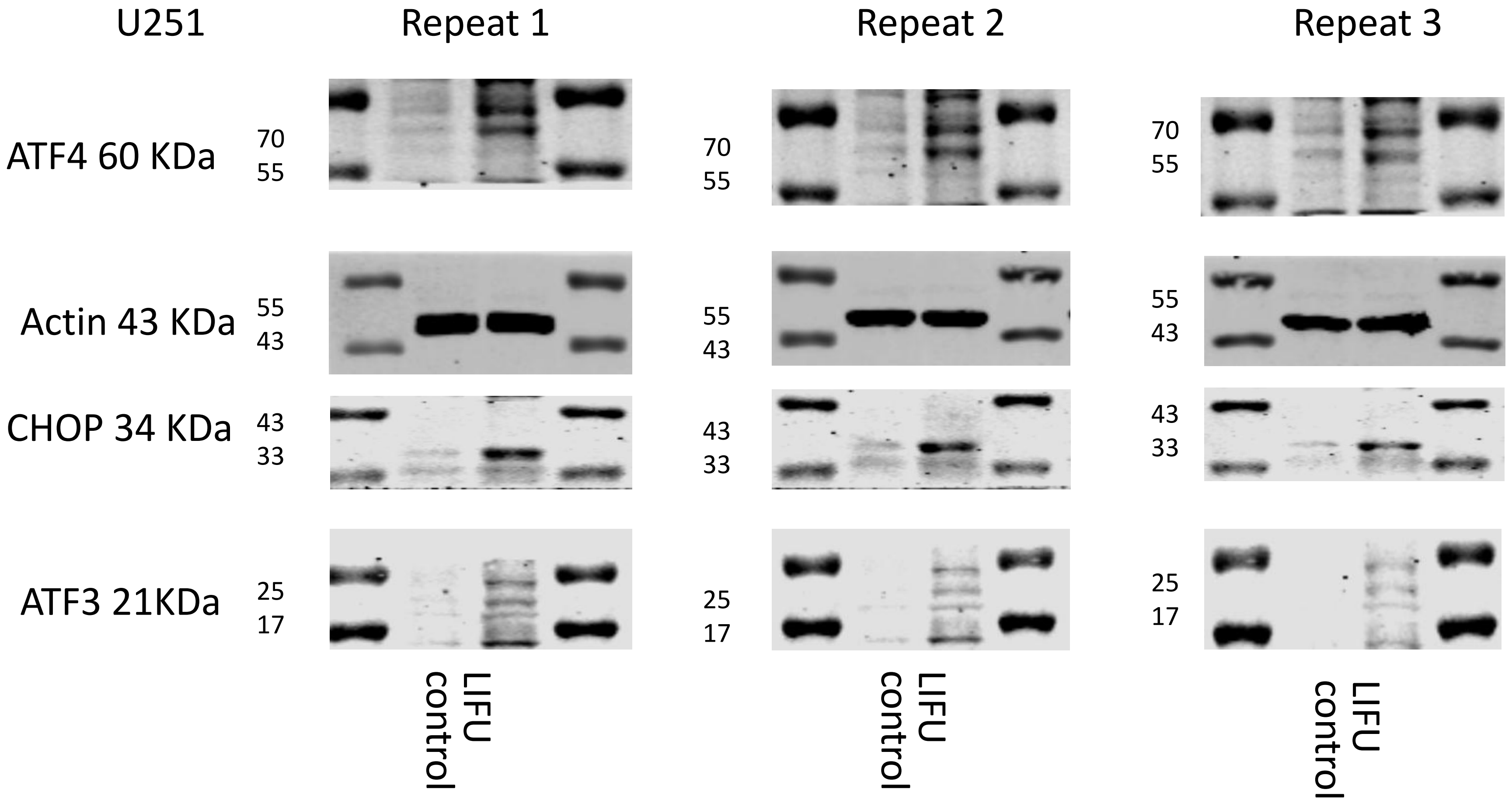
U251
Repeat 1
Repeat 2
Repeat 3
ATF4 60 KDa
Actin 43 KDa
CHOP 34 KDa
ATF3 21KDa
70
55
55
43
43
33
25
17
control
LIFU

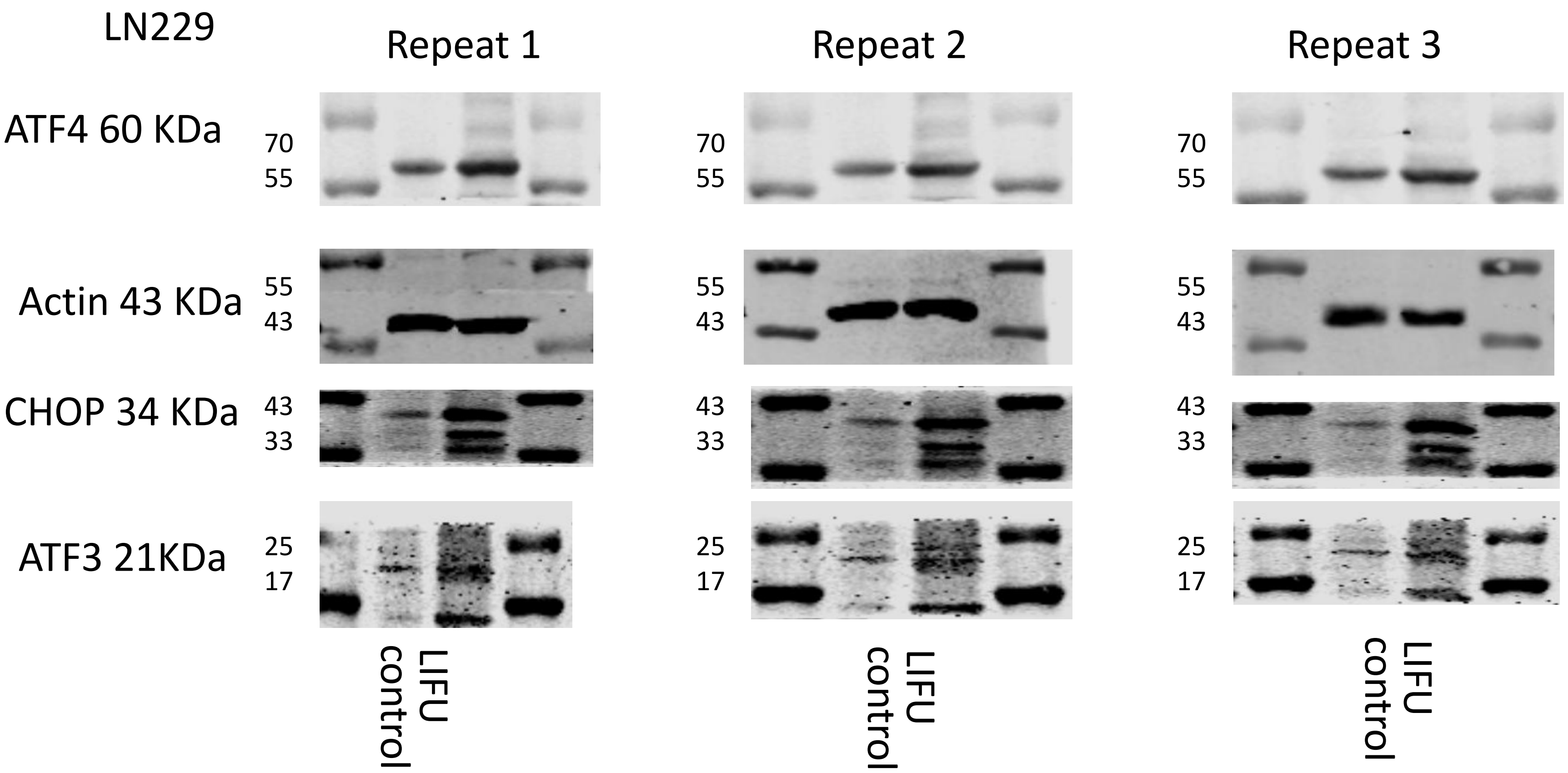
LN229
Repeat 1
Repeat 2
Repeat 3
ATF4 60 KDa
Actin 43 KDa
CHOP 34 KDa
ATF3 21KDa
70
55
55
43
43
33
25
17
70
55
55
43
43
33
25
17
70
55
55
43
43
33
25
17
control
LIFU
control
LIFU
control
LIFU

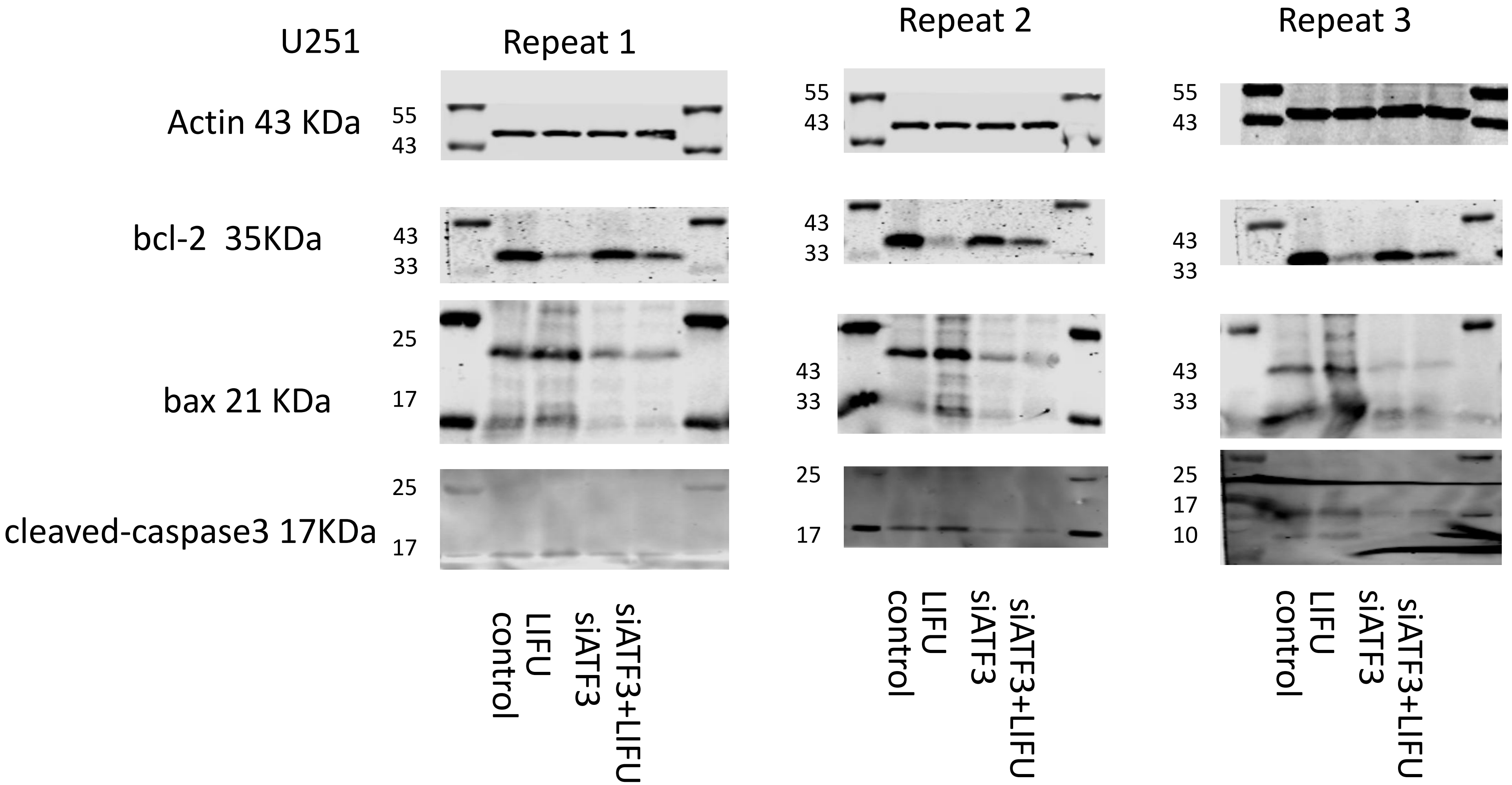
U251
Repeat 1
Repeat 2
Repeat 3
Actin 43 KDa
bcl-2 35KDa
bax 21 KDa
cleaved-caspase3 17KDa
55
43
33
25
17
10
control
LIFU
siATF3
siATF3+LIFU

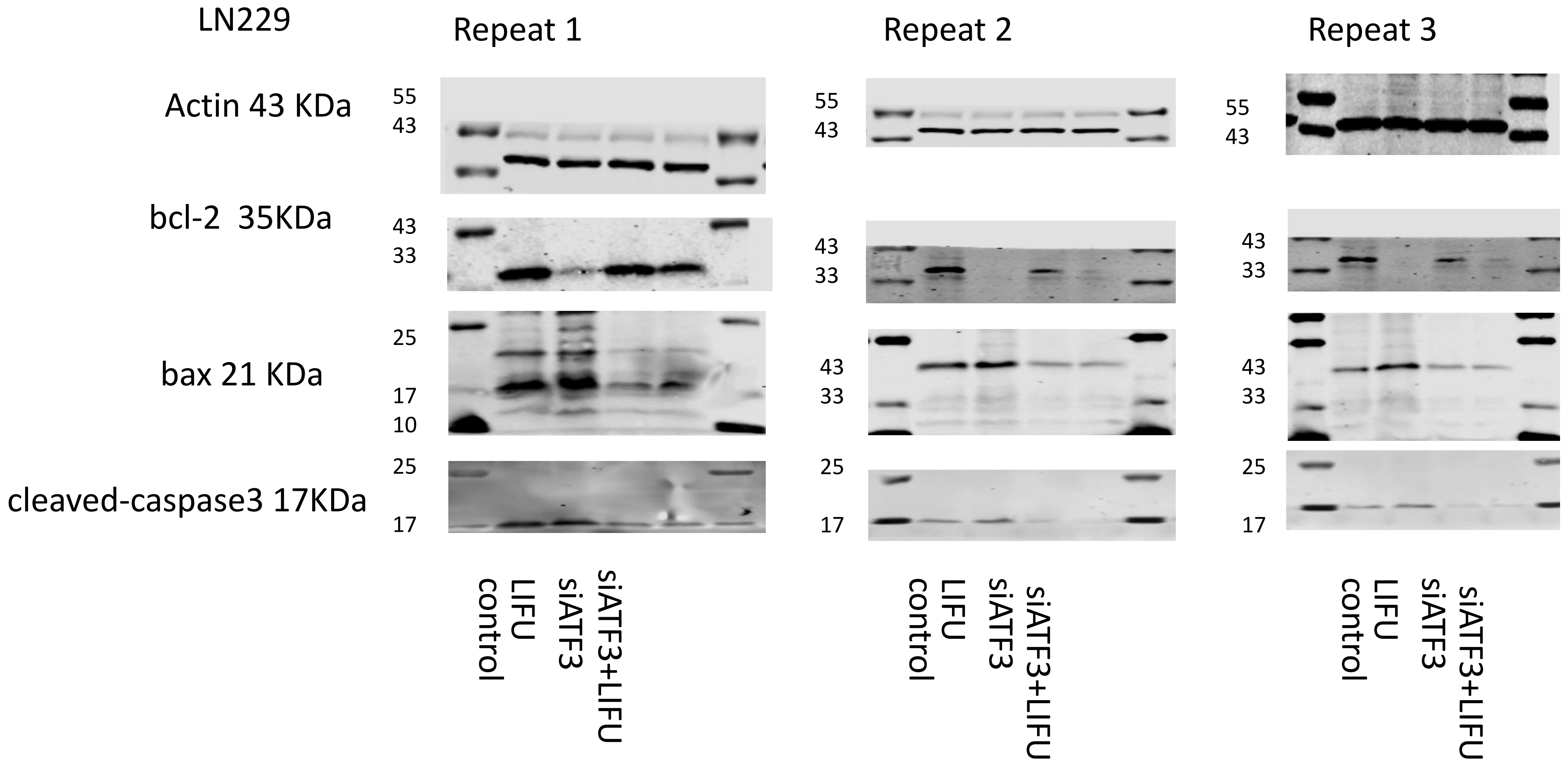
LN229
Repeat 1
Repeat 2
Repeat 3
Actin 43 KDa
bcl-2 35KDa
bax 21 KDa
cleaved-caspase3 17KDa
55
43
33
25
17
10
control
LIFU
siATF3
siATF3+LIFU

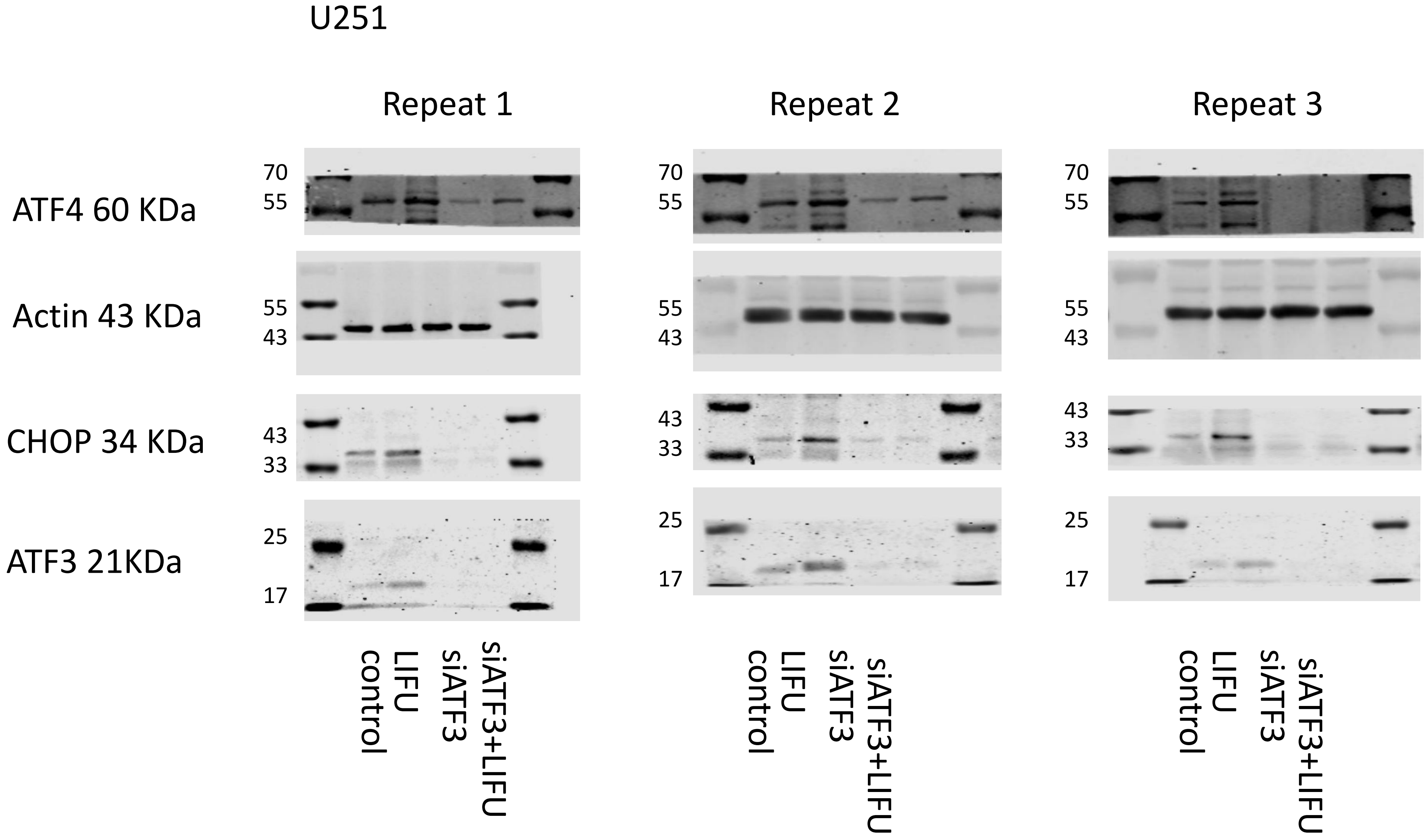
U251
Repeat 1
Repeat 2
Repeat 3
ATF4 60 KDa
Actin 43 KDa
CHOP 34 KDa
ATF3 21KDa
70
55
43
33
25
17
control
LIFU
siATF3
siATF3+LIFU

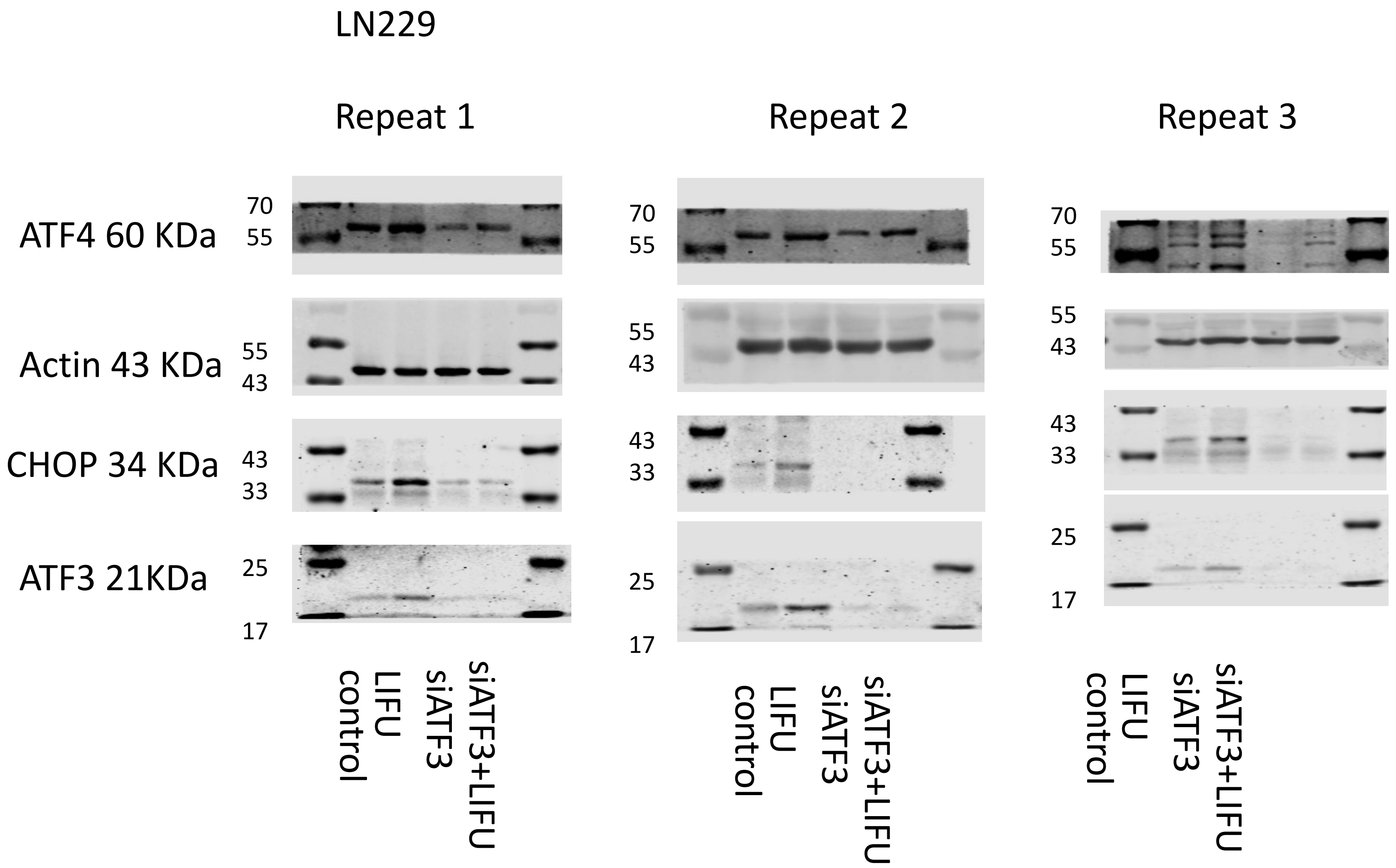

LN229
Repeat 1
Repeat 2
Repeat 3
ATF4 60 KDa
Actin 43 KDa
CHOP 34 KDa
ATF3 21KDa
70
55
55
43
43
33
25
17
control
LIFU
siATF3
siATF3+LIFU

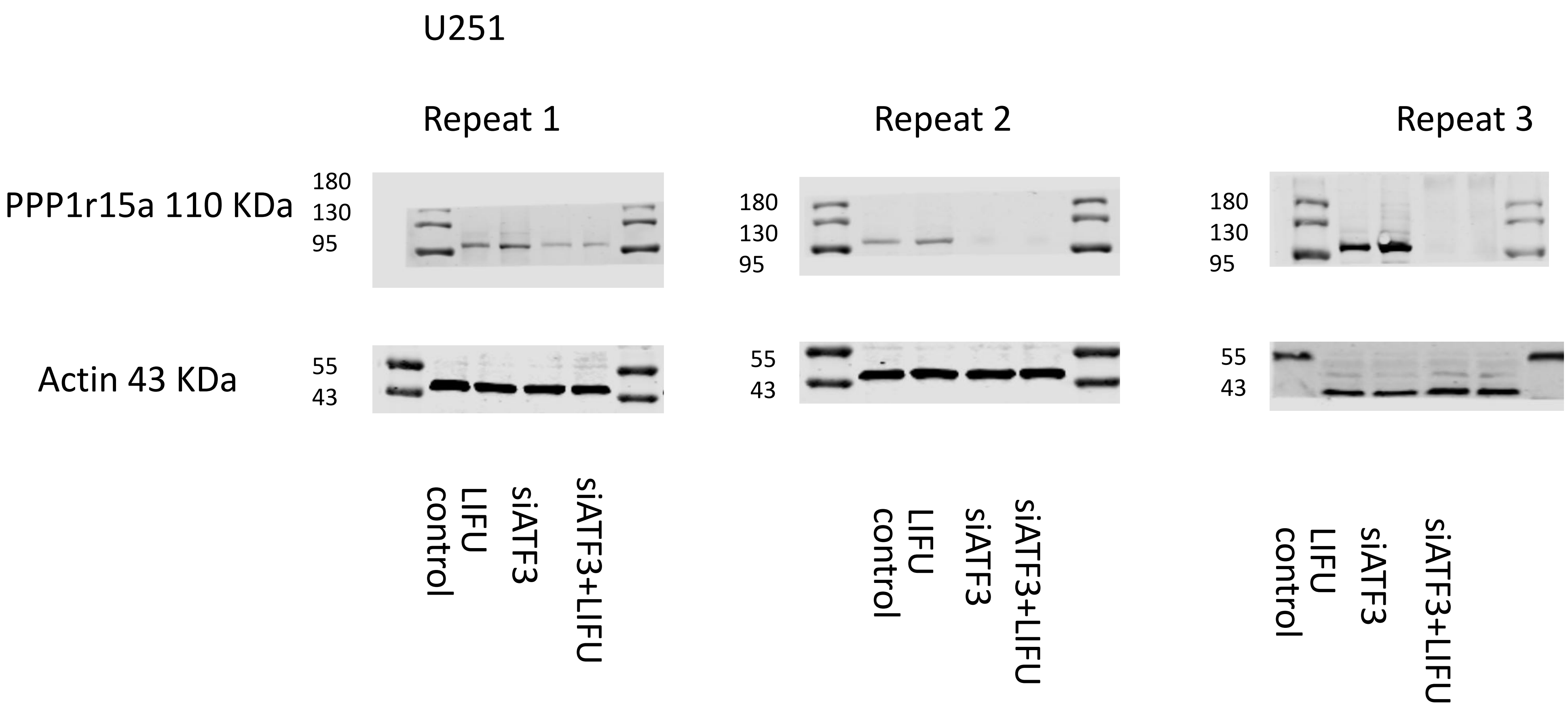
U251
Repeat 1
Repeat 2
Repeat 3
PPP1r15a 110 KDa
Actin 43 KDa
180
130
95
55
43
control
LIFU
siATF3
siATF3+LIFU

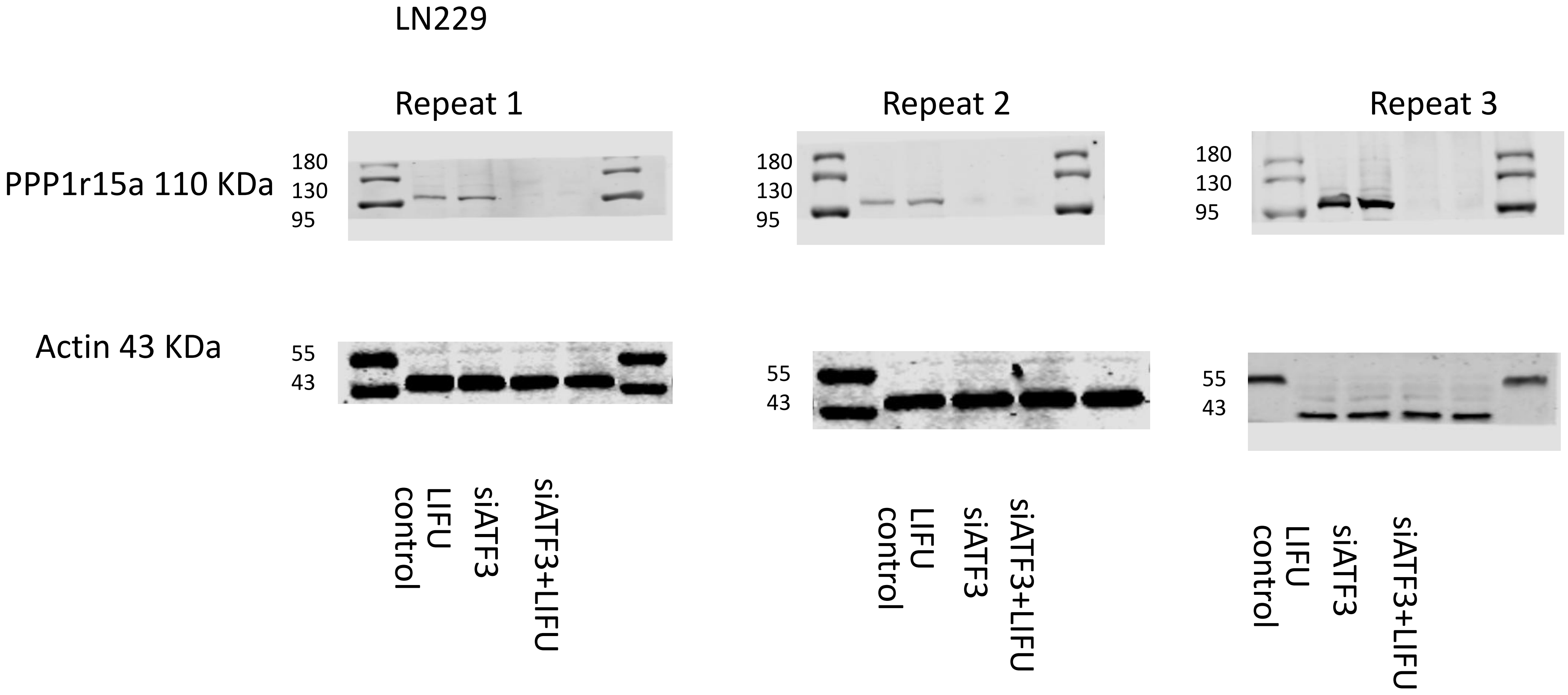

LN229
Repeat 1
Repeat 2
Repeat 3
PPP1r15a 110 KDa
Actin 43 KDa
180
130
95
55
43
control
LIFU
siATF3
siATF3+LIFU

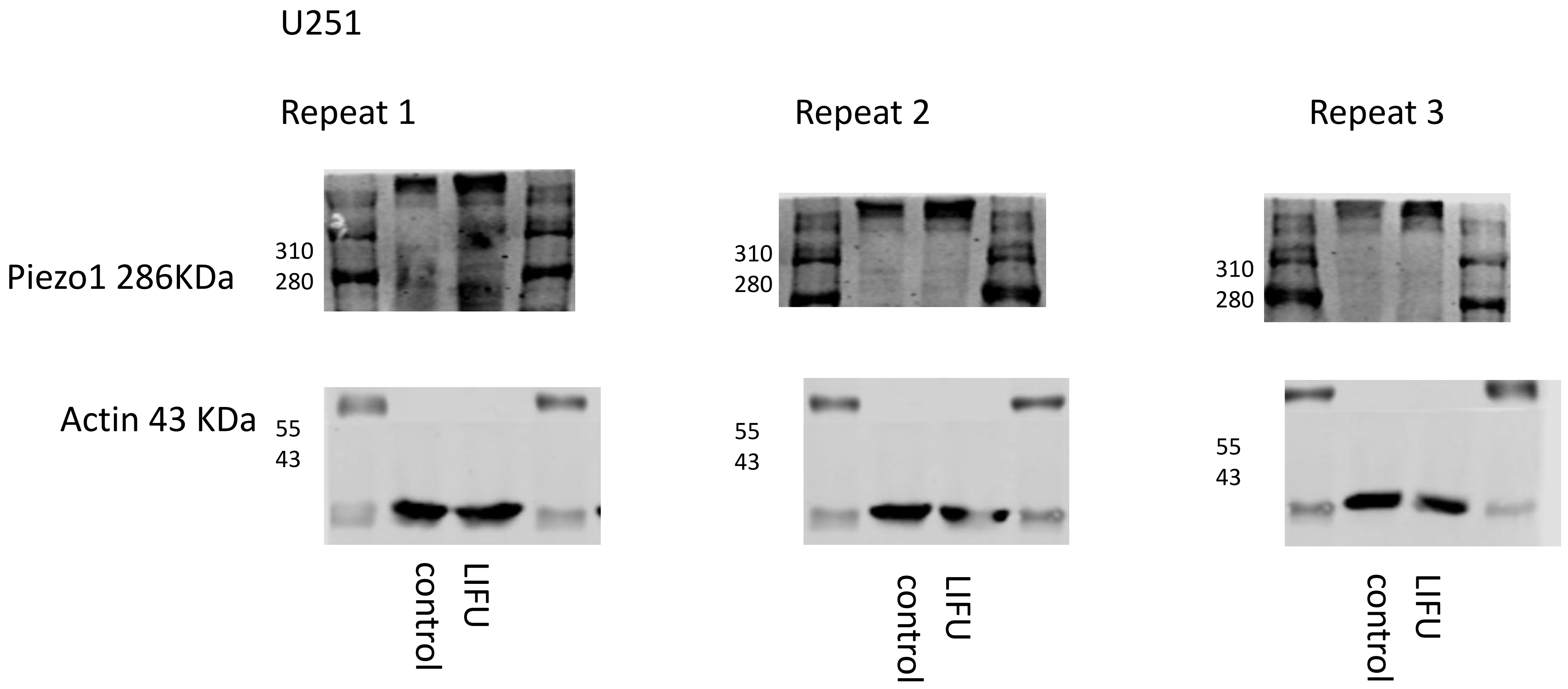
U251
Repeat 1
Repeat 2
Repeat 3
Piezo1 286KDa
Actin 43 KDa
310
280
310
280
310
280
55
43
55
43
55
43
control
LIFU
control
LIFU
control
LIFU

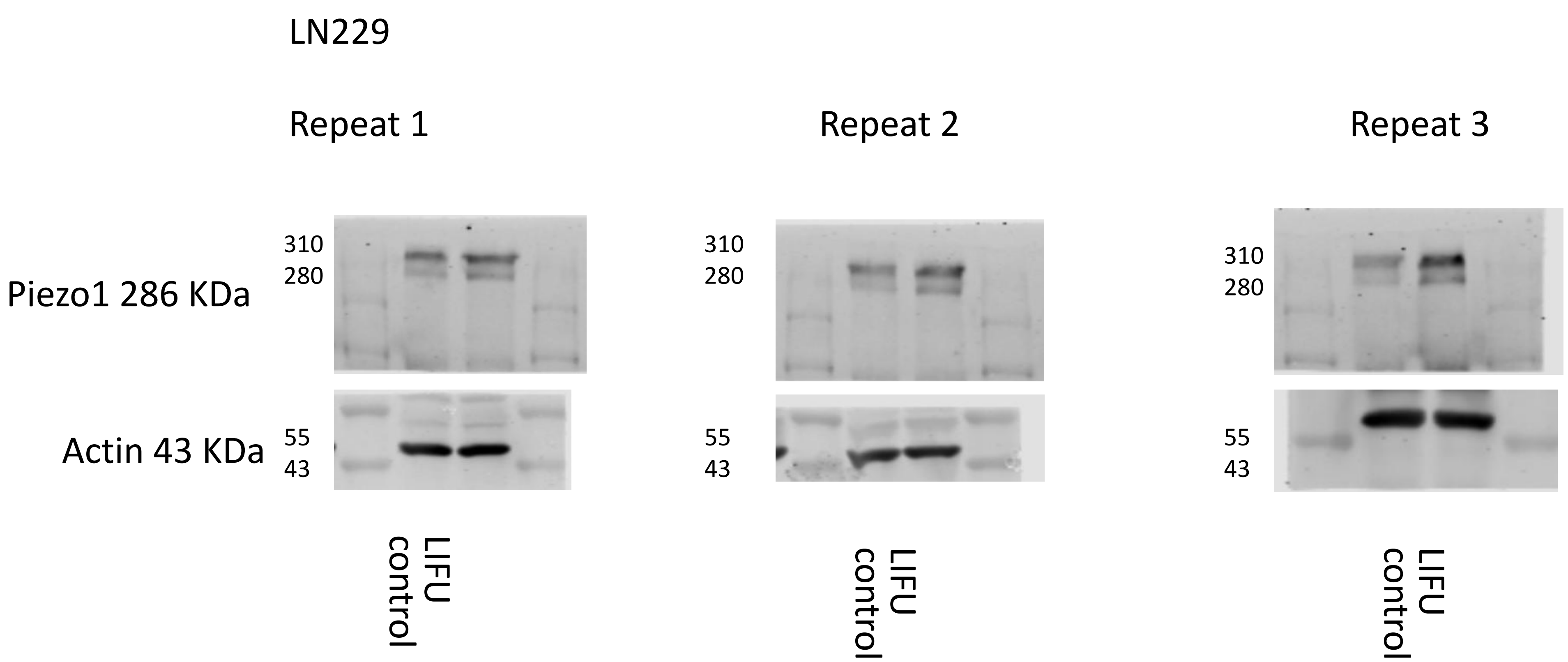

LN229
Repeat 1
Repeat 2
Repeat 3
Piezo1 286 KDa
Actin 43 KDa
310
280
55
43
LIFU
control

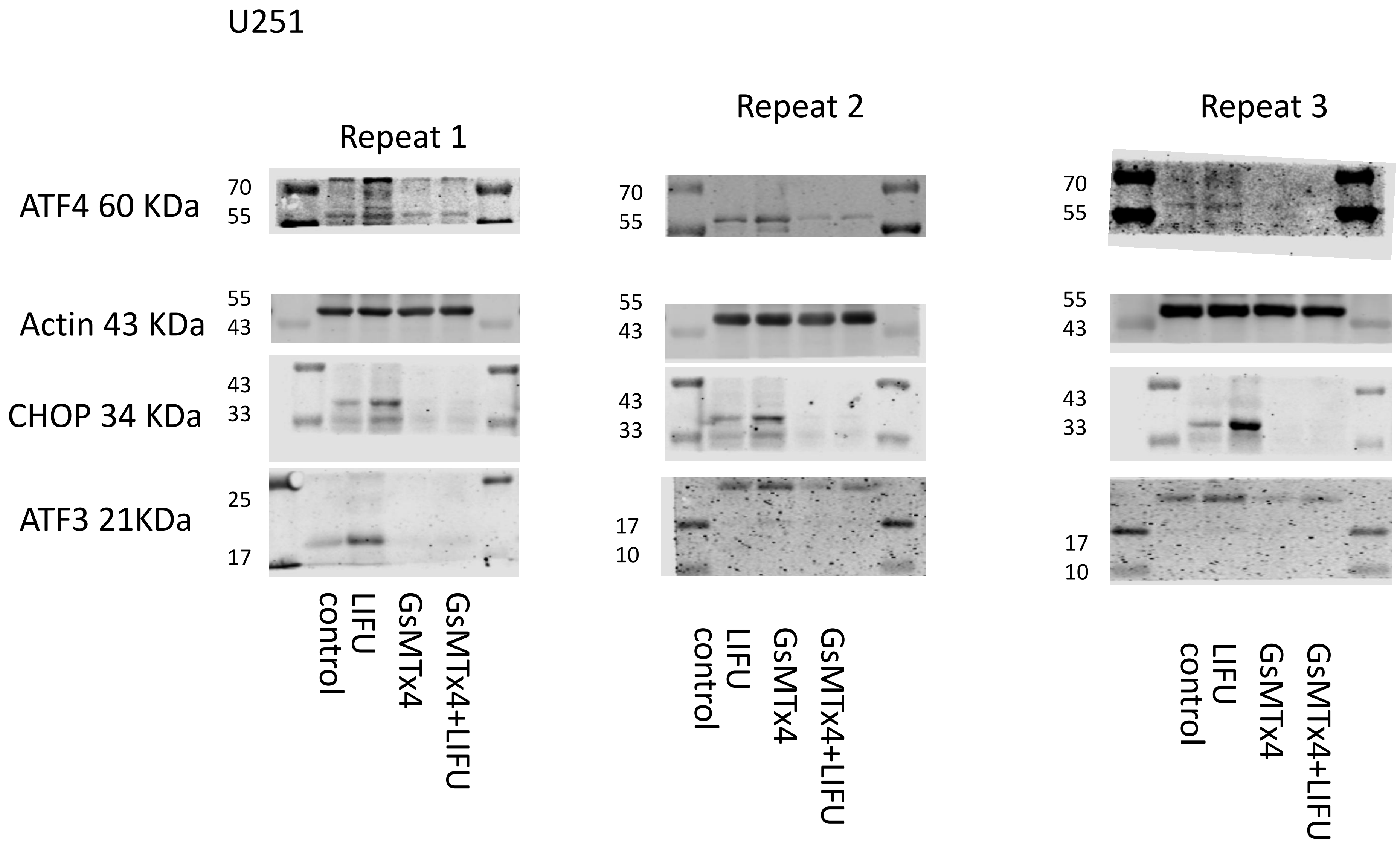
U251
Repeat 1
Repeat 2
Repeat 3
ATF4 60 KDa
Actin 43 KDa
CHOP 34 KDa
ATF3 21KDa
70
55
43
33
25
17
10
control
LIFU
GsMTx4
GsMTx4+LIFU

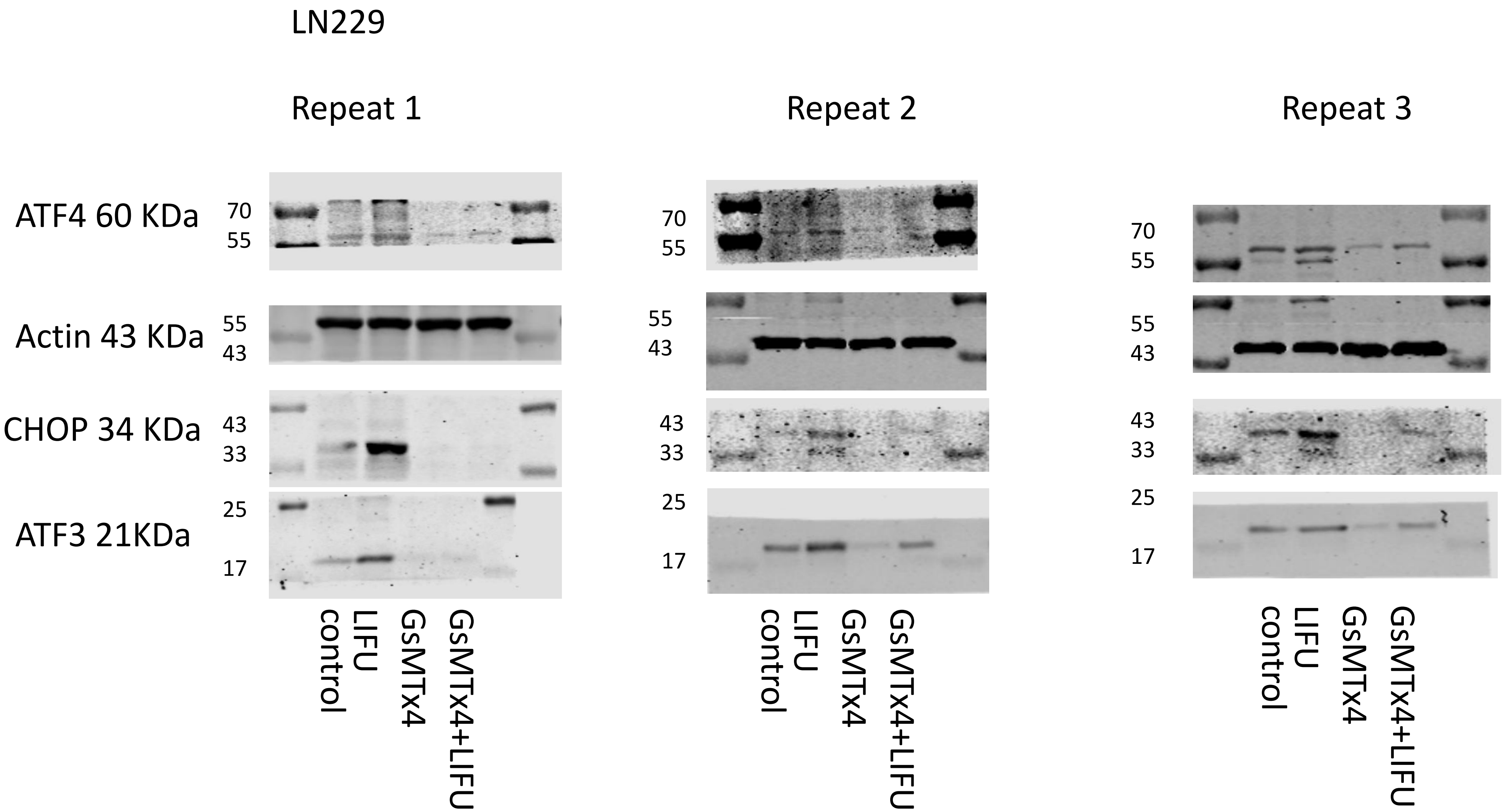
LN229
Repeat 1
Repeat 2
Repeat 3
ATF4 60 KDa
Actin 43 KDa
CHOP 34 KDa
ATF3 21KDa
70
55
43
33
25
17
control
LIFU
GsMTx4
GsMTx4+LIFU

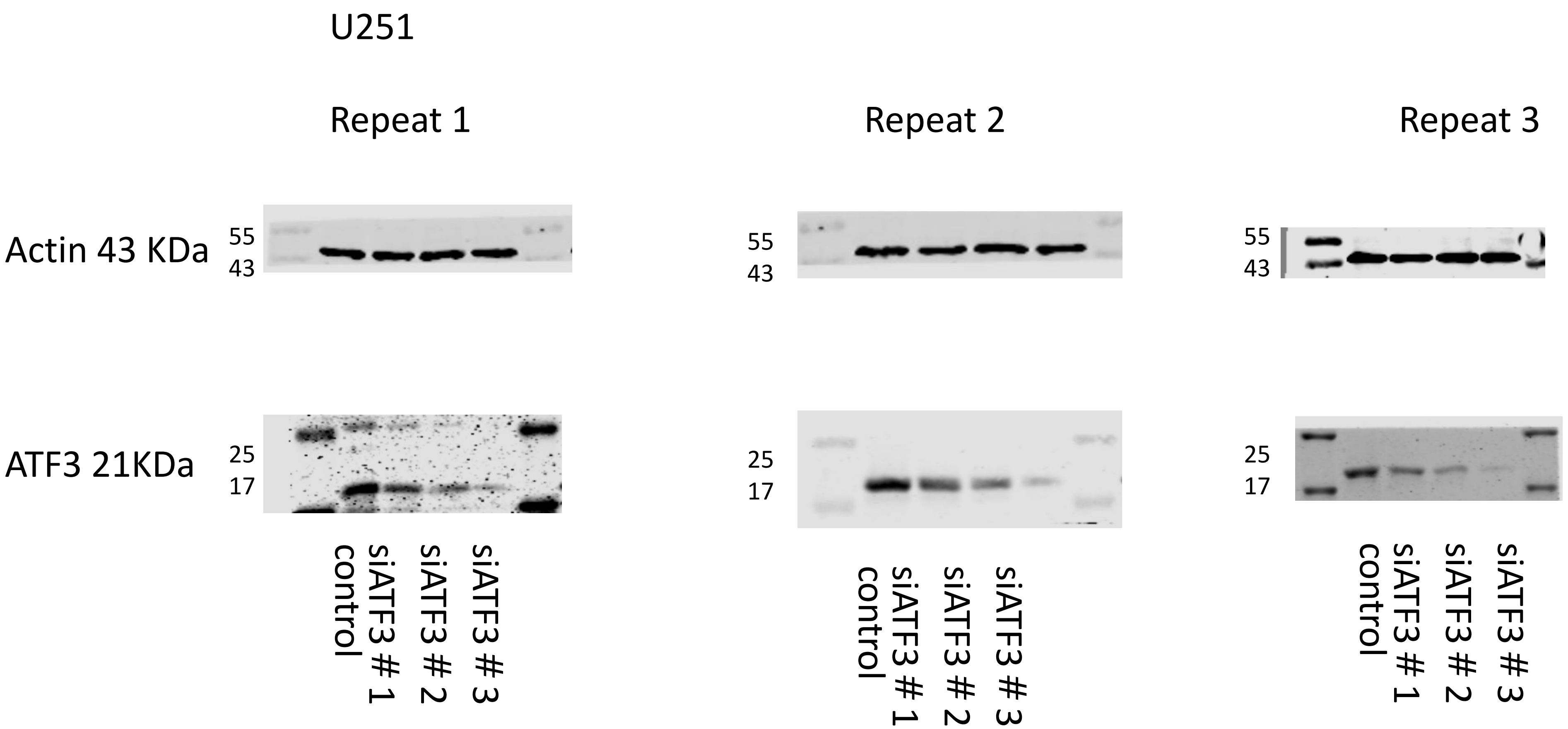
U251
Repeat 1
Repeat 2
Repeat 3
Actin 43 KDa
ATF3 21KDa
55
43
25
17
control
siATF3 # 1
siATF3 # 2
siATF3 # 3

LN229

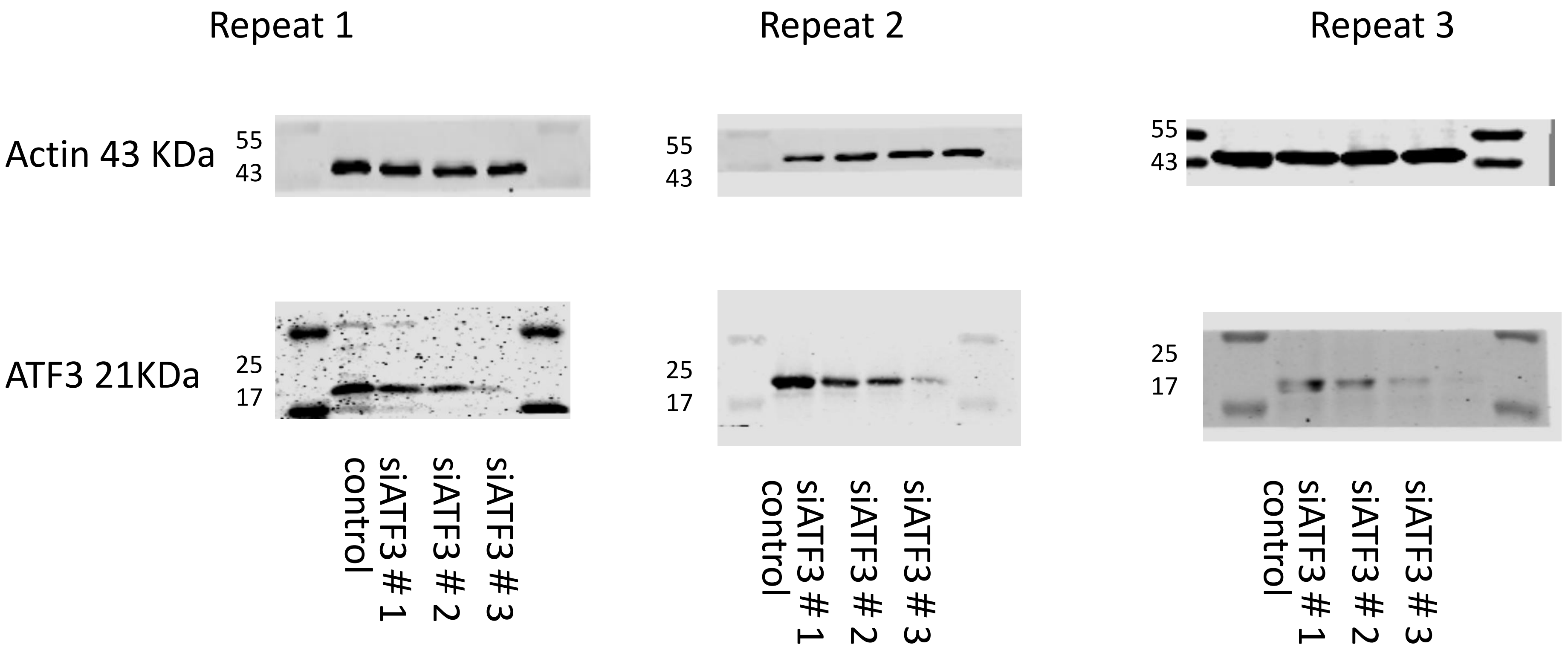
Repeat 1
Repeat 2
Repeat 3
Actin 43 KDa
ATF3 21KDa
55
43
25
17
control
siATF3 # 1
siATF3 # 2
siATF3 # 3

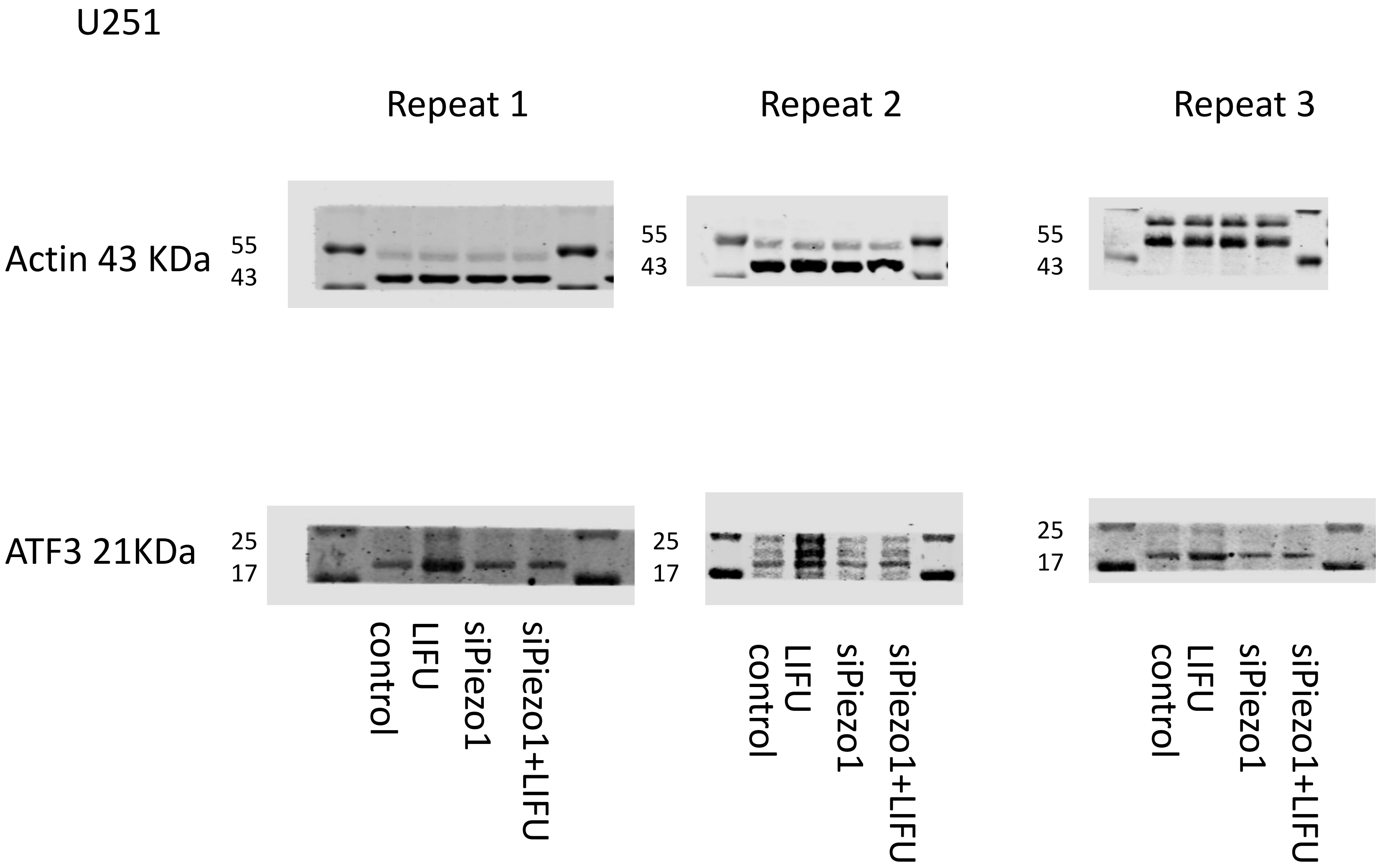
U251
Repeat 1
Repeat 2
Repeat 3
Actin 43 KDa
55
43
ATF3 21KDa
25
17
control
LIFU
siPiezo1
siPiezo1+LIFU

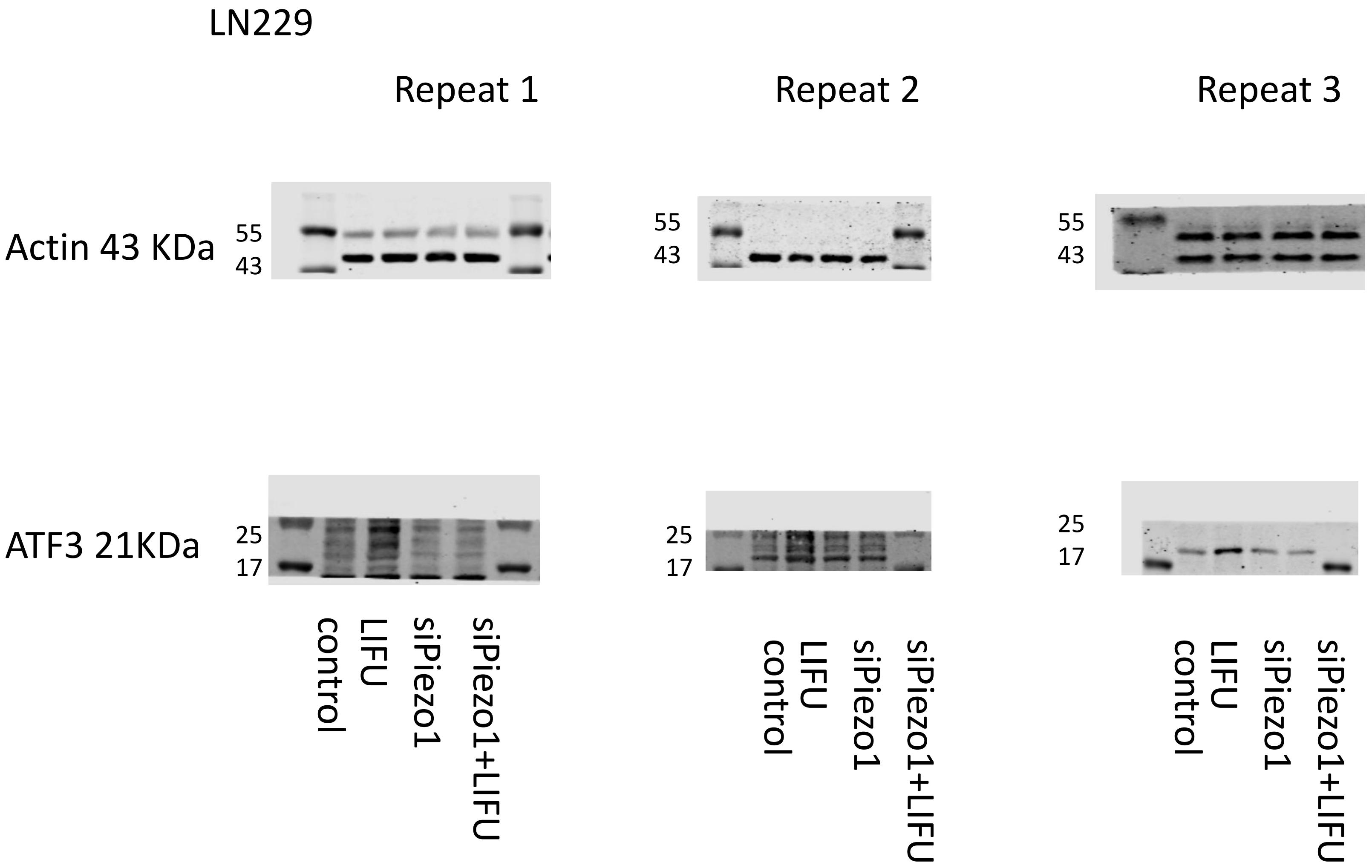

LN229
Repeat 1
Repeat 2
Repeat 3
Actin 43 KDa
ATF3 21KDa
55
43
25
17
control
LIFU
siPiezo1
siPiezo1+LIFU

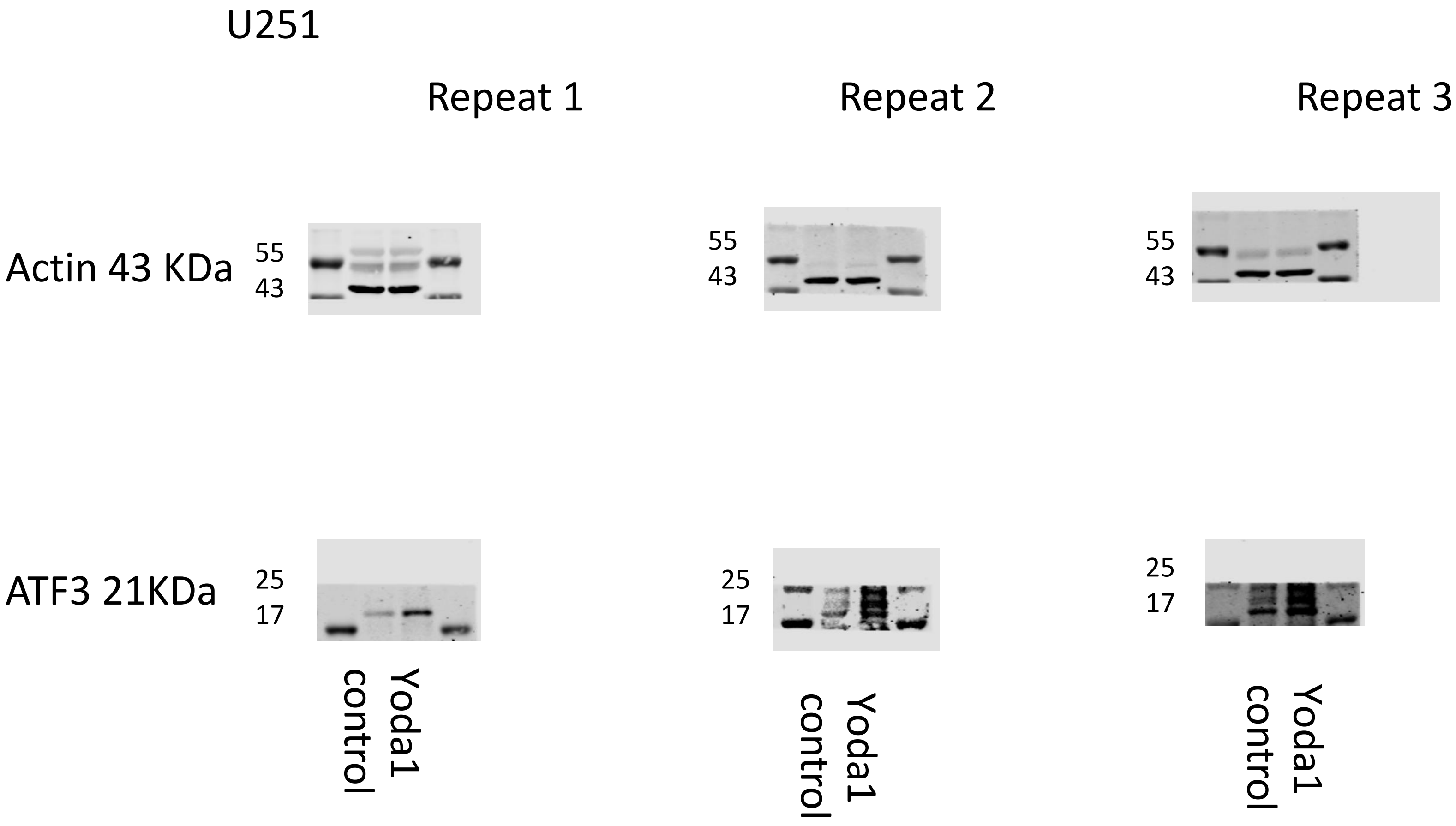
U251
Repeat 1
Repeat 2
Repeat 3
Actin 43 KDa
55
43
55
43
55
43
ATF3 21KDa
25
17
25
17
25
17
control
Yoda1
control
Yoda1
control
Yoda1

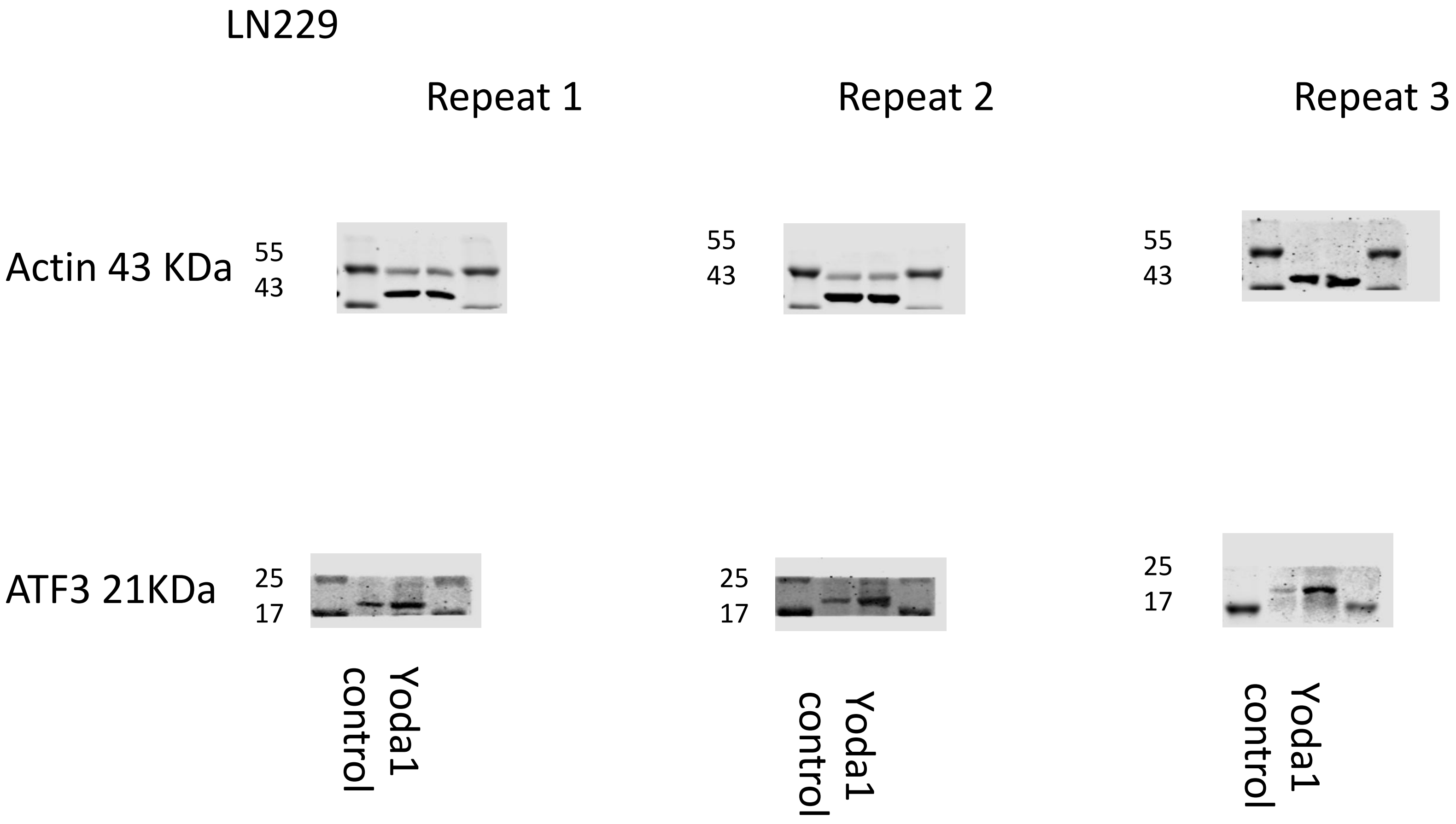
LN229
Repeat 1
Repeat 2
Repeat 3
Actin 43 KDa
55
43
55
43
55
43
ATF3 21KDa
25
17
25
17
25
17
control
Yoda1
control
Yoda1
control
Yoda1

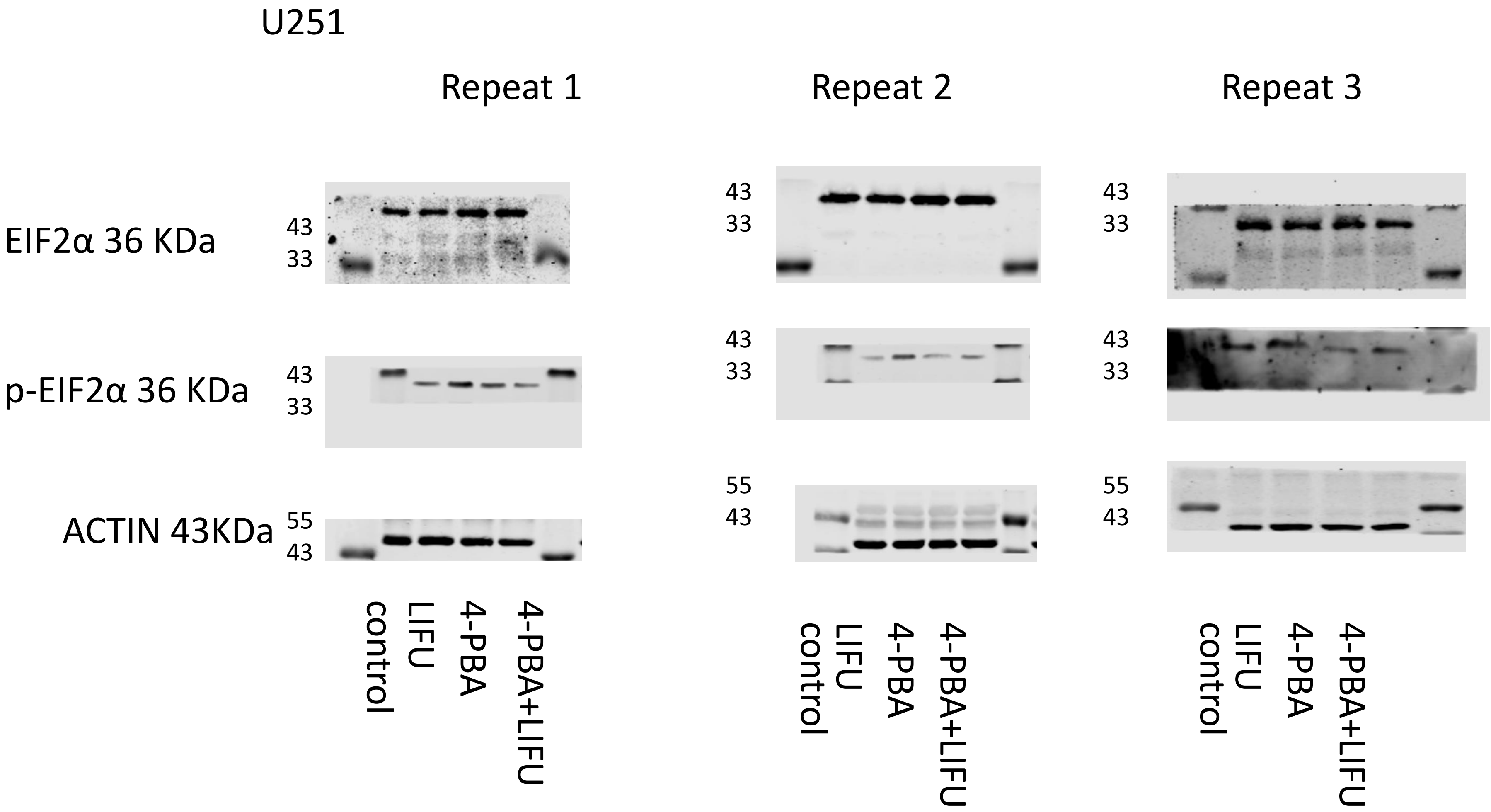
U251
Repeat 1
Repeat 2
Repeat 3
EIF2α 36 KDa
p-EIF2α 36 KDa
ACTIN 43KDa
43
33
55
control
LIFU
4-PBA
4-PBA+LIFU

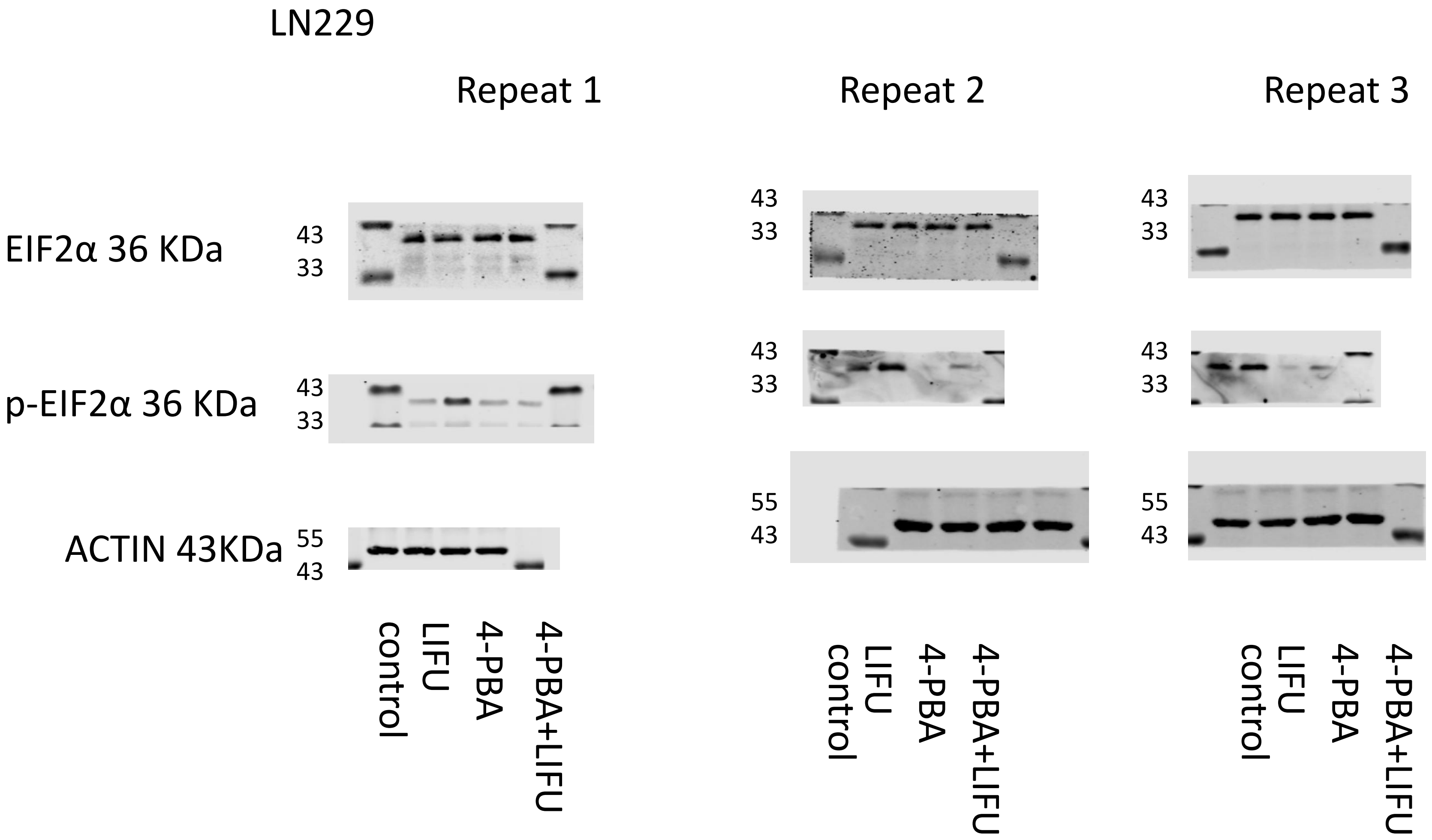

LN229
Repeat 1
Repeat 2
Repeat 3
EIF2α 36 KDa
p-EIF2α 36 KDa
ACTIN 43KDa
43
33
55
control
LIFU
4-PBA
4-PBA+LIFU
